# SpectralGuard: a Spectrally grounded λ-SHAP framework for XAI validity certification in pharmaceutical supply chain networks

**DOI:** 10.3389/frai.2026.1884880

**Published:** 2026-10-23

**Authors:** Ntebogang Dinah Moroke, Mmapula Merriam Lebele

**Affiliations:** North-West University, Potchefstroom, South Africa

**Keywords:** explainable AI, Fiedler eigenvalue, hypergraph Laplacian, pharmaceutical supply chain, South Africa, spectral governance, XAI validity certification, λ-SHAP

## Abstract

Permutation-based explainability methods such as SHAP are central to regulatory compliance in AI-governed pharmaceutical supply chains across Sub-Saharan Africa, yet their validity depends on a structural property that existing frameworks do not monitor: the algebraic connectivity of the supply hypergraph, quantified by the Fiedler eigenvalue λ_2_. We prove that λ_2_ → 0 is a sufficient condition for permutation-based XAI failure (Theorem 3.5): no increase in sampling effort can recover validity once network connectivity collapses. We introduce SpectralGuard, demonstrated on a calibrated synthetic DGP driven by real Eskom load-shedding and SARB ZAR/USD records (*n* = 3, 227 business days, 2014–2026); all spectral results are from the synthetic hypergraph. λ-SHAP shrinks attributions toward a fluid-regime prior using λ_2_(*t*) as the shrinkage weight, maintaining auditability at an explicit bias–variance cost (Remark 3.12). A probabilistic validity certificate *V*(*t*) combines four spectral metrics with SHA-256 tamper-evidence. An oracle experiment (*B* = 500, Seed = 2,026) demonstrates 70.4–89.2% attribution MSE reduction across governance regimes. The spectral gate is the decisive ablation component. In the synthetic DGP, early-warning detection leads classical baselines by 19 business days (λ_*c*_ alert preceding the λ_*m*_ crossing). Field validation on real CCMDD procurement data is planned for 2027 (PLMIF-2). A nine-step DHIS2 integration pathway supports SDG 3, 9, 13, and 16.

## 1 Introduction

Artificial intelligence is transforming the pharmaceutical supply chain management across Sub-Saharan Africa, where machine learning models now govern demand forecasting, inventory optimization, and procurement decisions for programmes serving tens of millions of chronic disease patients (Narayana et al., 2014; Nguyen et al., 2022; Dey et al., 2024; Ivanov et al., 2020; Saberi et al., 2019). At the center of these systems is an explainability requirement: procurement officers must justify AI recommendations to regulatory auditors using attribution methods such as SHAP, and those attributions must be valid at the moment they are acted upon. Whether that condition is satisfied depends not on the choice of attribution method, but on the structural state of the supply network itself, a fact that existing governance frameworks do not measure and cannot detect.

The COVID-19 pandemic exposed the fragility of this assumption at scale (Salehi et al., 2026). During the March–June 2020 lockdown, a major African chronic medicines distribution programme produced internally contradictory XAI explanations that raised concerns under Good Distribution Practice audit requirements (National Health Department, 2021). Procurement officers could not justify AI recommendations to regulators, yet the AI system appeared to be performing correctly by every standard metric. The failure was not algorithmic but geometric: the supply network had undergone a structural change that violated the conditions under which explainability methods are theoretically valid, and no existing framework detected this before it propagated into patient access failures.

Existing approaches to XAI governance in regulated supply chains address this problem at the wrong level (Rudin et al., 2019; Covert et al., 2021). Standard tools (MAPE thresholds, SHAP drift monitoring, permutation importance variance) are all *post-hoc* diagnostics that identify explanation instability only after it has already corrupted procurement decisions (Lundberg et al., 2017). Graph neural network explainers (Ying et al., 2019) improve attribution accuracy on pairwise graph representations, but cannot recover the joint accountability structure of multi-party pharmaceutical supply contracts, which bind manufacturer, logistics operator, and dispensing cluster under a single regulatory hyperedge (Chan et al., 2018). The core theoretical question these approaches leave unanswered is: *what structural condition of the supply network is necessary and sufficient for any explainability method to produce valid attributions?*

### 1.1 The central question

This study asks: can a pharmaceutical AI governance system be *theoretically grounded* (with a provable validity certificate for its explanations), *structurally aware* (encoding the network geometry that governs explanation validity), *self-correcting* (producing auditable attributions even under network stress), and *integration-ready* (architected for existing national health digital infrastructure with a costed proof-of-concept pathway pending field validation)? SpectralGuard and λ-SHAP provide the theoretical and computational foundations for this.

### 1.2 Interdisciplinary framing

This study occupies the intersection of three fields that have developed largely in parallel. *Spectral hypergraph theory* (Chung et al., 1997; Zhou et al., 2007) provides the Fiedler eigenvalue λ_2_ of the normalized hypergraph Laplacian and the Cheeger inequality that bounds network connectivity. *Explainable AI (XAI) theory* (Lundberg et al., 2017; Rudin et al., 2019) provides the axiomatic foundations of additive attribution methods and the consistency conditions under which they are theoretically valid in networked AI systems. *Pharmaceutical supply chain management* (Narayana et al., 2014; National Health Department, 2021) provides the operational context, the regulatory requirements, and the South African chronic medicines distribution programme as a natural laboratory for stress testing the framework. The novelty of this study is the disciplined integration of all three: spectral theory governs when XAI is valid, XAI theory defines what validity means, and supply chain management defines what auditability requires in regulatory practice. Section 5 tests whether the integration is load-bearing by demonstrating the explanation validity collapses entirely when the spectral condition is violated: an ablation of the geometry, not the algorithm.

The framework is *spectrally grounded*: the algebraic connectivity λ_2_, mixing time *T*_rel_ = 2/λ_2_ (Levin and Peres, 2017), Cheeger conductance (Louis, 2015), and Ollivier-Ricci curvature (Ollivier et al., 2009) are all structural quantities drawn from the physics of random processes on graphs (Newman et al., 2018; Chan et al., 2018). λ-SHAP uses λ_2_(*t*) as the *spectrally derived* shrinkage coefficient: the weight is exactly the quantity that bounds the attribution error in Theorem 3.6, making the correction structure-derived, not empirically tuned (Rudin et al., 2019). Three structural properties of pharmaceutical distribution networks anchor this spectral framing. First, supply contracts bind multiple parties simultaneously under a single regulatory obligation (National Health Department, 2021): a manufacturer, a cold-chain logistics operator, and a dispensing cluster form a *hyperedge*, not a pairwise edge, and standard graph representations lose the joint accountability structure governing both network connectivity and attribution validity. Second, the mixing time of the *lazy random walk* *P* = *I*−**L**/2 on the hypergraph satisfies *T*_rel_ = 2/λ_2_ (since *P* = *I*−**L**/2 gives μ_2_ = 1−λ_2_/2, so *T*_rel_ = 1/(1−μ_2_) = 2/λ_2_; see Levin and Peres (2017) Chapter 12): when λ_2_ declines, the AI system cannot collect globally representative feature distributions within any finite decision horizon, violating the consistency condition of additive XAI methods (Theorem 3.6). Third, documented infrastructure shocks in the South African context; load-shedding and currency volatility, produce precisely the class of exogenous hyperedge weight perturbations that drive λ_2_ toward zero, motivating the empirical validation design and grounding the theoretical framework in operationally realistic disruption scenarios.

This study makes five contributions to the XAI and pharmaceutical AI governance literature:

1. Spectral XAI validity theorem. We prove that λ_2_ → 0 is a sufficient condition for failure of *any* permutation-based XAI method (SHAP, LIME, Kernel SHAP) in a hypergraph-structured AI system, providing the first theoretically grounded validity certificate for XAI in network-governed AI systems. Integrated Gradients, as a path-based rather than permutation-based method, lies outside the theorem's formal scope (see Remark 3.5).
2. λ-SHAP: spectral shrinkage estimator. We introduce λ-SHAP, which uses λ_2_(*t*) as a shrinkage weight between the current SHAP estimates and a validated fluid-regime prior. The core shrinkage idea extends prior work on regularized attribution (Lundberg et al., 2017; Rudin et al., 2019); the novelty is using λ_2_ as the weight, grounding the shrinkage in spectral network theory rather than statistical regularization.
3. Probabilistic XAI validity certificate. A continuous validity score *V*(*t*)∈[0, 1] derived from four spectral governance metrics via logistic regression, with dynamic threshold adaptation and SHA-256 tamper-evidence. The probabilistic certification layer extends standard binary governance flags; the novelty is the λ_2_-grounded certificate structure, not the logistic regression itself.
4. Four spectral governance metrics. SGM, EDI, TSI, and ORC with analytically derived or Monte Carlo-validated null distributions, operationalizing the spectral validity condition for daily procurement governance.
5. Proxy-panel calibration and proof-of-concept deployment pathway. Proxy calibration on a cleaned panel of real Eskom load-shedding and SARB exchange rate data spanning the 2014–2026 period, and a nine-step costed integration pathway (R915,000) describing how the framework would be operationalized within existing DHIS2 national health infrastructure pending field validation (PLMIF-2, 2027).

Explicit novelties: (i) To our knowledge, the first proof that the Fiedler eigenvalue governs validity of the general class of permutation-based XAI methods; (ii) To our knowledge, the first spectral XAI attribution method (λ-SHAP) with a provable network validity certificate; (iii) To our knowledge, the first probabilistic XAI validity certification system for pharmaceutical AI governance, calibrated using *n* = 3, 227 days of real Eskom and SARB shock data driving a synthetic DGP; (iv) A demonstration (in a calibrated synthetic DGP driven by real South African infrastructure data) that XAI collapse is a geometric rather than algorithmic failure mode; (v) The first open governance dataset pairing synthetic pharmaceutical hypergraph dynamics with publicly available infrastructure shock series (data available at https://doi.org/10.5281/zenodo.20330335 (v.01), https://doi.org/10.5281/zenodo.22730264 (v.02), https://doi.org/10.5281/zenodo.22745540 (v.03).

The remainder proceeds as follows. Section 2 reviews related work. Section 3 develops the theoretical framework. Section 4 presents the data and methods. Section 5 reports all results.

Section 6 discusses implications and concludes.

## 2 Literature review

### 2.1 AI-governed pharmaceutical supply chains

Pharmaceutical supply chains encompass the full value chain from raw material to patient, and their governance encompasses operational continuity, equitable access, and regulatory accountability (Narayana et al., 2014). The complexity of pharmaceutical distribution, including fluctuating demand, cold-chain dependencies, and multi-tier logistics structures, creates governance demands that conventional management approaches cannot meet (Ivanov et al., 2020; Saberi et al., 2019). AI and ML paradigms address these complexities through predictive analytics, enhanced decision-making, and optimized resource allocation (Dey et al., 2024; Ivanov et al., 2020; Saberi et al., 2019; Lim et al., 2021). Salehi et al. (2026) show that adaptive AI strategies were essential for the supply chain integrity during COVID-19, while Kosasih and Brintrup (2022) shows that graph-based AI models improve disruption prediction accuracy by 31% over traditional time-series methods. Yet across this literature, a critical gap persists: none of these studies provides a real-time, mathematically grounded mechanism for certifying when AI explanations are theoretically valid and when they are not. This article addresses that gap.

### 2.2 XAI theory, SHAP validity, and certification

The governance of AI in regulated industries requires explainable, auditable, and documented recommendations (Rudin et al., 2019). SHAP values (Shapley, 1953; Lundberg et al., 2017) satisfy four axiomatic properties: *efficiency, symmetry, dummy*, and *additivity*. Under regularity conditions, the SHAP estimator is asymptotically unbiased:


$$\begin{array}{l} E\left[{\hat{\phi }}_{i}\right]\to \phi_{i}^{*}\;\text{as }n\to \infty , \end{array}$$ (1)

where $\phi_{i}^{*}$ is the true Shapley value (Lundberg et al., 2017). However, Covert et al. (2021) demonstrate that this convergence requires a stationary data-generating process: when the underlying network structure changes, the explanatory subspace rotates, violating Equation 1.

GNN explainers (Ying et al., 2019; Wasi et al., 2024) have been proposed as governance tools, but their reliance on pairwise graph reductions create false attribution pathways under network stress (Kosasih and Brintrup, 2022). (Kosasih and Brintrup 2022) show that GNN-based supply chain models exhibit explanation drift in 38% of disruption scenarios. These failures motivate a structural rather than algorithmic approach to XAI governance, one that certifies the *conditions* under which attributions are generated, not merely the attributions themselves. Recent studies on XAI governance requirements in regulated environments Bhatt et al., (2020) establishes measurable standards for auditable attribution methods in deployment, confirming the regulatory demand that this study addresses.

The mathematical properties of SHAP that make it regulatorily attractive also make it structurally vulnerable to network topology changes. When the network enters topological arrest, SHAP estimates attributions from a locally trapped distribution, violating the additivity axiom in practice, even while satisfying it formally (Covert et al., 2021). This distinction between formal and practical validity is the theoretical heart of the XAI certification problem this article addresses.

Recent methodological advances in robust attribution have addressed related but distinct challenges. *Conditional SHAP* (Aas et al., 2021) computes attributions by marginalizing over feature distributions conditioned on observed feature values, improving accuracy when features are correlated. *Causal SHAP* (Lundberg et al., 2017) incorporates causal structure into the marginalization to avoid spurious attributions from non-causal feature associations. *Robust SHAP* (Covert et al., 2021) provides a unified framework for removal-based attribution that encompasses both conditional and marginal SHAP as special cases; (Bhatt et al. 2020) establish governance requirements for XAI in deployed systems, identifying stability and auditability as the two properties most critical for regulated environments; and Ancona et al. (2019) show that gradient-based attribution methods share structural assumptions that make them susceptible to the same distributional failures as permutation-based methods. Under network connectivity loss, however, the conditioning sets themselves become poorly estimated locally trapped distributions, so conditional SHAP and robust attribution methods inherit the same mixing-time vulnerability as standard SHAP (Table 1, 12–18% reduction vs. λ-SHAP's 71–90%). SpectralGuard addresses a complementary and logically prior problem: not the quality of an attribution estimate conditional on a valid distribution, but whether a valid distribution exists in the first place. While robust attribution methods address instability conditional on having a representative background, SpectralGuard certifies whether the background itself is representative, a necessary precondition that no existing method monitors.

**Table 1:** Ablation study: MSE against known true Shapley values (*B* = 500, Seed = 2,026, linear oracle *n* = 10, background corruption: $x_{\text{bg}}\sim U\left[0,1-{\left(1-\lambda_{2}\right)}^{1.5}\right]$, same model as Table 5).

Config.	Active	Green (0.50)	Amber (0.28)	Red (0.08)
(a) Full λ-SHAP	Gate+prior+*V*(*t*)	0.237 ± 0.010	0.117 ± 0.003	0.093 ± 0.001
(b) No gate (λ_2_≡1)	None	0.829 ± 0.046	0.888 ± 0.050	0.940 ± 0.053
(c) No *V*(*t*) cert^*^	Gate+prior	0.237 ± 0.010	0.117 ± 0.003	0.093 ± 0.001
(d) Standard SHAP	None	0.829 ± 0.046	0.888 ± 0.050	0.940 ± 0.053
(e) Cond. SHAP^†^	Conditioning	0.022 ± 0.000	0.019 ± 0.000	0.021 ± 0.000
*Reduction vs. (d)*				
(a) Full λ-SHAP		71.4%	86.8%	90.1%
(b) No gate		0.0%	0.0%	0.0%
(e) Cond. SHAP		97.3%	97.9%	97.8%

^*^ *V*(*t*) provides probabilistic confidence intervals and SHA-256 tamper-evidence; it is a governance output layer, not an estimation component. Configurations (a) and (c) are attribution-equivalent, confirming that all MSE gain derives from the spectral gate. ^†^ Conditional SHAP conditions each feature's background on a ±0.10 window around *x*_*i*_; non-conditioned features still use the corrupted background, limiting overall gain. MSE values differ in scale from Table 5 because the ablation prior is set to the exact population mean of the oracle DGP (${\bar{\phi }}_{i}^{\text{fluid}}=0$, the true $E\left[\phi_{i}^{*}\right]$) rather than a sample-estimated fluid mean; reduction percentages are directionally consistent with oracle results: Full λ-SHAP achieves 71.4%–90.1% reduction; conditional SHAP achieves 97.3%–97.8% reduction under oracle conditions (known DGP, correct conditioning) but this advantage narrows substantially under real-world misspecification (SR_λ_ = 0.79 vs. 0.94). SR_λ_ (stability robustness, real proxy panel): full λ-SHAP = 0.94; standard SHAP = 0.61 (Table 6).

Standard pairwise graph representations are structurally inadequate for pharmaceutical distribution, where multi-party supply contracts bind manufacturers, logistics operators, and dispensing facilities under a single regulatory corridor (Hearnshaw et al., 2013; Chan et al., 2018).

### 2.3 Hypergraph theory and spectral methods in supply chain AI

Standard pairwise graph representations are structurally inadequate for pharmaceutical distribution, where multi-party supply contracts bind manufacturers, logistics operators, and dispensing facilities under a single regulatory corridor (Hearnshaw et al., 2013; Chan et al., 2018). A hyperedge e∈E in a pharmaceutical supply network has cardinality |*e*|≥3 by regulatory definition (National Health Department, 2021): every supply contract involves at minimum a supplier, a logistics operator, and a dispensing cluster. Projecting such contracts onto pairwise edges destroys the joint accountability structure and creates spurious independence assumptions that degrade both network metrics and XAI attribution quality (Zhou et al., 2007).

The normalized hypergraph Laplacian (Chung et al., 1997; Zhou et al., 2007; Chan et al., 2018; Zhu and Segarra, 2023) provides a spectral characterization of network connectivity whose second smallest eigenvalue λ_2_ governs information propagation and mixing time. Louis (2015) establishes the hypergraph Cheeger inequality:


$$\begin{array}{l} \frac{\lambda_{2}}{2}\leq \Phi \left(H\right)\leq \sqrt{2\lambda_{2}}, \end{array}$$ (2)

bounding λ_2_ from above and below by the minimum normalized cut Φ(H). Persistent homology (Zhu and Segarra, 2023) provides a complementary topological characterization of network resilience through the Topological Stability Index, capturing alternative supply paths that absorb node-level disruptions and detecting path loss before connectivity loss, the mechanism underlying the early detection advantage reported in Section 5.4. The connection between spectral methods and XAI validity in networked AI systems has not been established in the prior literature; this article provides that link through Theorem 3.6.

### 2.4 Research gaps and positioning

Four specific research gaps motivate this article. *Gap 1:* The absence of a provably valid XAI method that remains auditable under network stress; existing frameworks monitor outputs, not structural validity conditions (Covert et al., 2021; Rudin et al., 2019). *Gap 2:* The absence of a correct structural representation of multi-party supply networks, where pairwise graph representations can lose joint failure topology in multi-party contracts (Kosasih and Brintrup, 2022). *Gap 3:* The absence of an operationally deployable governance framework linking mathematical theory to regulatory audit requirements (Rudin et al., 2019). *Gap 4:* The absence of a probabilistic validity certificate with confidence quantification for XAI in pharmaceutical AI systems. All existing governance systems produce binary or ternary pass/fail classifications; none provides a continuous validity score with posterior confidence intervals, dynamic threshold adaptation, or a tamper-evident cryptographic audit record. The study occupies the intersection of five thematic clusters in the literature: Network Theory, AI Governance, Pharmaceutical Supply Chain, Disruption Management, and Sustainability.

## 3 Theoretical framework

### 3.1 Hypergraph representation

** Definition 3.1** (Weighted Distribution Hypergraph). *Let* H=(V,E,W) *where* $V=\left\{v_{1},\ldots ,v_{n}\right\}$ *is the set of* *n* *distribution nodes*; $E=\left\{e_{1},\ldots ,e_{m}\right\}$ *is the set of* *m* *hyperedges representing multi-party supply contracts; and* **W** = diag(*w*_1_, …, *w*_*m*_) *with* *w*_*k*_ *proportional to daily dispensing throughput. The incidence matrix* **H**∈{0, 1}^*n*×*m*^ *has* *H*_*vk*_ = 1 if *v*∈*e*_*k*_, 0 *otherwise*.

** Definition 3.2** (Normalized Hypergraph Laplacian). *Let* ${\text{D}}_{v}=\mathrm{diag}\left(\underset{k}{\sum }w_{k}H_{vk}\right)$ *and* **D**_*e*_ = diag(|*e*_*k*_|). *The normalized hypergraph Laplacian is*:


$$\begin{array}{l} \text{L}=\text{I}-{\text{D}}_{v}^{-1/2}\text{H}\text{W}{\text{D}}_{e}^{-1}{\text{H}}^{⊤}{\text{D}}_{v}^{-1/2}, \end{array}$$ (3)

*Note (Path B): Equation 3 is the edge-degree-normalized clique expansion of Zhou et al. (2007): each hyperedge *e*_*k*_ is replaced by a clique with edge weights *w*_*k*_/|*e*_*k*_|. SpectralGuard does *not* claim to avoid clique expansion; the contribution is monitoring λ_2_(*t*) continuously as a leading governance signal. With mean hyperedge cardinality $\bar{\left|e\right|}=2.34$, most contracts are near-bilateral, so the practical difference between this operator and a non-reducible alternative is small in the current DGP. with spectrum 0 = λ_1_ ≤ λ_2_ ≤ ⋯ ≤ λ_*n*_ ≤ 2 (Chan et al., 2018; Zhou et al., 2007). *Note on spectral bound*: the [0, 2] bound follows from Chan et al. (2018) Theorem 1.1 for the normalized hypergraph Laplacian with edge-degree normalization **D**_*e*_ = diag(|*e*_*k*_|); Chung (Chung et al., 1997) establishes the analogous bound for graph Laplacians only. The Fiedler eigenvalue λ_2_ governs mixing time via Equation 4.*

### 3.2 Information processing theory

Information Processing Theory (IPT) (Galbraith, 1974; Tushman and Nadler, 1978) predicts that ML explanations become unreliable when the network's information propagation capacity is insufficient for global feature attribution. The mixing time *T*_rel_ quantifies this capacity:


$$\begin{array}{l} T_{\text{rel}}\leq \frac{1}{\lambda_{2}}\log\left(\frac{2\sqrt{\text{vol}\left(H\right)}}{\varepsilon }\right), \end{array}$$ (4)

so *T*_rel_ → ∞ as λ_2_ → 0. IPT predicts governance failure when information processing demand Ω(*t*) exceeds capacity Γ(λ_2_(*t*)) = κ_max_·λ_2_(*t*), motivating:

H_1_ (IPT). *r*(λ_2_, −EDI)>0 with *p* < 0.05, confirming that the spectral gap decline drives the explanation drift.

### 3.3 Complexity theory

Complexity Theory (Scheffer et al., 2009; Gao et al., 2022) predicts discontinuous performance degradation near critical thresholds through critical slowing down (Scheffer et al., 2009). The Ollivier-Ricci curvature provides the spatial counterpart:


$$\begin{array}{c} \mathrm{Pr}\left(\text{cascade}|\kappa \left(e,t\right)<\kappa_{\text{crit}}\right) \end{array}$$

$$\begin{array}{c} >\mathrm{Pr}\left(\text{cascade}|\kappa \left(e,t\right)\geq \kappa_{\text{crit}}\right) \end{array}$$ (5)

where κ_crit_ = −0.20, (Equation 5) motivating:

H_2_ (CT). Spectral governance metrics provide statistically earlier disruption detection than classical and GNN baselines, consistent with complexity theory's prediction that geometric precursors anticipate scalar performance degradation.

### 3.4 Spectral XAI validity theorem

The IPT and CT frameworks converge on a single mathematical object: the Fiedler eigenvalue λ_2_. The following theorem generalizes this to the entire class of permutation-based XAI methods.

** Remark 3.3** (Assumption on background sourcing.). *Assumption 1 (Network-local background). Background samples for* Φ∈P *at time* *t* *are restricted to the connected component of the query node in* H(t), *because feature observations are sourced from procurement transactions, and disconnected nodes stop transacting. This is the link between the mixing time and SHAP background sampling: the empirical feature distribution is supported on the supply network, so drawing background samples is equivalent to a random walk on the connectivity structure; as λ_2_ → 0, that walk cannot mix to stationarity within any finite decision horizon. Under this assumption Theorem 3.6 holds for all* Φ∈P. *If a fixed external background is available, Assumption 1 does not apply*.

** Definition 3.4** (Permutation-based attribution.). *An attribution method* Φ∈P *is* *permutation-based* *if its estimator* ${\hat{\phi }}_{i}$ *is computed by marginalizing* *f*(**x**) *over permutations of feature subsets drawn from the empirical distribution* ${\hat{P}}_{V}$ *of supply network* H. *The class* P *includes SHAP (Lundberg et al., 2017), LIME (Ribeiro et al., 2016), and Kernel SHAP.*

** Remark 3.5** (Integrated gradients and the theorem scope.). *Integrated Gradients (IG) (Sundararajan et al., 2017) is *not* a permutation-based method in the sense of Definition 3.4: IG computes attributions by integrating the gradient of *f* along a path from a baseline input **x**′ to the query input **x**, without marginalizing over permutations of the empirical distribution. Accordingly, IG is excluded from the formal class P and from the scope of Theorem 3.6.*

*However, IG remains susceptible to a related but distinct failure mode under network connectivity loss. When* λ_2_ → 0, *the baseline* **x**′ *(typically the zero vector or the feature mean) becomes unrepresentative of the true supply-network marginal distribution: the integration path traverses a region of input space that is inconsistent with the global network geometry. The resulting attribution errors are not governed by the mixing-time bound of Theorem 3.6 but rather by the distributional discrepancy between the baseline and the global mean under topological arrest. A formal extension of the theorem to path-based attribution methods under network stress is identified as future theoretical work; the present article's formal results are restricted to* P.

** Theorem 3.6** (Spectral XAI inconsistency: general class.). *Let* Φ∈P *be applied to a Lipschitz model* *f* (||*f*||_Lip_ < ∞) *on* H *with Fiedler eigenvalue* λ_2_. *As* λ_2_ → 0, *let* S⊂V *denote the connected component containing the query node, and let* π(S)=vol(S)/vol(H)<1 *(proper subset under topological arrest). There exists a class of Lipschitz models* *f* *and features* *i* *satisfying* *c*(1−π(*S*))||*f*||_Lip_>0 (*verified when* π(*S*) < 1 *and* ||*f*||_Lip_>0) *for which*:


$$\begin{array}{l} \underset{N\to \infty }{\mathrm{liminf}}E\left||{\hat{\phi }}_{i}^{\left(t\right)}-\phi_{i}^{*}\right|{|}_{L_{1}};\geq ;c\left(1-\pi \left(S\right)\right)||f|{|}_{\text{Lip}};>;0, \end{array}$$ (6)

*uniformly in *N*. No increase in sampling effort can recover the true Shapley value: permutation-based estimators are *inconsistent* under topological arrest.*

*Proof*. We establish the bound in four steps.

Step 1 (Mixing time and distribution trapping). By Definition 3.4, every Φ∈P estimates *E*[*f*(**x**)∣**x**_*S*_] by marginalizing *f* over feature subsets drawn from the empirical supply-network distribution ${\hat{P}}_{V}$. The relaxation time of the lazy random walk on H satisfies *T*_rel_ = 2/λ_2_ (Levin and Peres, 2017) (since *P* = *I*−*L*/2 gives μ_2_ = 1−λ_2_/2, so *T*_rel_ = 1/(1−μ_2_) = 2/λ_2_). By Equation 4, the mixing time to ε-stationarity satisfies $T_{\text{mix}}\left(\varepsilon \right)\leq T_{\text{rel}}\log\left(2\sqrt{\text{vol}\left(H\right)}/\varepsilon \right)$. As λ_2_ → 0, *T*_mix_(ε) → ∞ for every fixed ε>0, so any finite decision horizon *T*_dec_ satisfies *T*_dec_<*T*_mix_(ε), and the walk has not reached stationarity. Consequently, ${\hat{P}}_{V}$ converges not to the global supply-network distribution $P_{V}$ but to a locally trapped measure ${\hat{P}}_{S}$ supported on the connected component containing the query point.

Step 2 (Total variation divergence). The Cheeger inequality (Equation 2) gives $\Phi \left(H\right)\leq \sqrt{2\lambda_{2}}$. When λ_2_ → 0, Φ(H)→0, meaning there exists a cut (*S, S*^*c*^) with |*E*(*S, S*^*c*^)|/min(vol(*S*), vol(*S*^*c*^)) → 0. The random walk probability of crossing from *S* to *S*^*c*^ within *T*_dec_ steps therefore vanishes, so $d_{\text{TV}}\left({\hat{P}}_{S},P_{V}\right)\to 1$ as λ_2_ → 0 (Levin and Peres, 2017).

Step 3 (Attribution error lower bound). For any Φ∈P, the attribution error satisfies:


$$\begin{array}{l} \left||{\hat{\phi }}_{i}-\phi_{i}^{*}\right|{|}_{L_{1}};\geq ;d_{\text{TV}}\left({\hat{P}}_{S},P_{V}\right)\cdot ||f|{|}_{\text{Lip}}. \end{array}$$ (7)

This follows from the dual representation of total variation: $d_{\text{TV}}\left(\mu ,\nu \right)=\underset{||g|{|}_{\text{Lip}}\leq 1}{\sup}|E_{\mu }\left[g\right]-E_{\nu }\left[g\right]|/2$, applied with *g* = *f* (appropriately normalized). The Shapley value $\phi_{i}^{*}$ integrates *f* over $P_{V}$, while ${\hat{\phi }}_{i}$ integrates over ${\hat{P}}_{S}$; the difference is bounded below by the left-hand side of Equation 7.

Step 4 (Deriving the 2/λ_2_ variance bound). We now connect the mixing-time argument to the stated bound $M_{\Phi }/\lambda_{2}^{\left(t\right)}$.

The spectral gap λ_2_ is the slowest non-zero decay rate of the Markov semigroup on H (Levin and Peres, 2017). After *T*_dec_ steps, the probability mass remaining in the trapped component *S* satisfies (Levin and Peres, 2017) (Equation 8):


$$\begin{array}{l} \mathrm{Pr}\left(X_{T_{\text{dec}}}\in S|X_{0}\in S\right);\geq ;e^{-\lambda_{2}T_{\text{dec}}}, \end{array}$$ (8)

so $d_{\text{TV}}\left({\hat{P}}_{S},P_{V}\right)\geq 1-e^{-\lambda_{2}T_{\text{dec}}}$. For small λ_2_ (i.e., λ_2_*T*_dec_≪1, the regime of interest when connectivity has collapsed), a first-order Taylor expansion gives (Equation 9):


$$\begin{array}{l} d_{\text{TV}}\left({\hat{P}}_{S},P_{V}\right);\geq ;1-e^{-\lambda_{2}T_{\text{dec}}};\approx ;\lambda_{2}T_{\text{dec}}. \end{array}$$ (9)

For the upper bound, each permutation drawn by Φ∈P samples from the trapped distribution ${\hat{P}}_{S}$. The effective number of independent permutation samples is bounded above by *T*_dec_/*T*_rel_ = λ_2_*T*_dec_/2 [since samples within *T*_rel_ = 2/λ_2_ steps are correlated (Levin and Peres, 2017)]. The variance of the estimator ${\hat{\phi }}_{i}$ is therefore inflated by a factor of *T*_rel_ = 2/λ_2_ relative to the i.i.d. case. With *C*_Φ_>0 a method-specific constant bounding the per-sample contribution, the variance inflation gives:


$$\begin{array}{l} E\left||{\hat{\phi }}_{i}^{\left(t\right)}-\phi_{i}^{*}\right|{|}_{L_{1}};\leq ;\frac{C_{\Phi }||f|{|}_{\text{Lip}}}{\lambda_{2}^{\left(t\right)}};=:;\frac{M_{\Phi }}{\lambda_{2}^{\left(t\right)}}, \end{array}$$ (10)

where *M*_Φ_: = *C*_Φ_||*f*||_Lip_>0 is finite for any fixed ||*f*||_Lip_ < ∞. As $\lambda_{2}^{\left(t\right)}\to 0$, this upper bound grows without bound; the lower bound converges to the finite positive constant *c*(1−π(*S*))||*f*||_Lip_>0, establishing inconsistency rather than divergence. As $d_{\text{TV}}\left({\hat{P}}_{S},P_{V}\right)\to 1-\pi \left(S\right)>0$, the lower bound converges to the finite positive constant *c*(1−π(*S*))||*f*||_Lip_>0. This constant is bounded away from zero *uniformly in* *N*: no increase in permutation samples eliminates this irreducible error floor, because ${\hat{P}}_{S}$ is structurally separated from $P_{V}$ regardless of sampling effort. The estimator is therefore *inconsistent* under topological arrest, establishing Equation 6. *Full mathematical expansions of all four steps, including the auxiliary Lemma 1.1, the complete total variation derivation (A1–A3 in* *Appendix 1**), and the treatment of the edge case* λ_2_ = 0*, are provided in Sections A1–A4 in* *Appendix 1*.

** Remark 3.7** (Sufficient condition only.). *Theorem 3.6 establishes that λ_2_ → 0 is a *sufficient* condition for XAI failure; it is **not** a necessary condition. Attribution methods can fail for other reasons (distributional shift, model misspecification, insufficient sample size) even when λ_2_ remains large. Conversely, some methods may exhibit partial robustness in specific structured settings, even under mild connectivity loss. The contribution of Theorem 3.6 is thus a *certificate direction*: λ_2_>λ_*c*_ certifies that connectivity-induced failure has not occurred; it does not preclude other failure modes. The validity certificate *V*(*t*) in Section 3.8 aggregates multiple governance metrics precisely because λ_2_ alone is not a complete validity criterion.*

** Corollary 3.8** (Method-agnostic validity certificate.). λ_2_(*t*)>λ_*c*_ *is a* *validity certificate* for any Φ∈P: *auditors certify attribution validity without inspecting attribution values or specifying which XAI method was used*.

** Lemma 3.9** (Davis-Kahan governance bound (Davis, 1965; Yu et al., 2015).). *Let* **L** *be the normalized hypergraph Laplacian (Definition 3.2) and* $\tilde{\text{L}}=\text{L}+\text{E}$ *a perturbed version with perturbation matrix* **E**. *Let* V *and* $\tilde{V}$ *denote the eigenvector matrices of* **L** *and* $\tilde{\text{L}}$ *respectively, and let* $\Theta \left(V,\tilde{V}\right)$ *denote the matrix of principal angles between their column spaces. Provided* λ_2_>||**E**||_2_ *(eigengap condition) (Equation 11):*


$$\begin{array}{l} \left||\sin\Theta \left(V,\tilde{V}\right)\right|{|}_{F}\leq \frac{||\text{E}|{|}_{F}}{\lambda_{2}-||\text{E}|{|}_{2}}. \end{array}$$ (11)

*In the Red regime, the eigengap condition fails and no SHAP subspace stability bound exists.*

The GPD-spectral tail coupling theorem (Theorem 2.1) is stated and proved in full in Appendix 2, including the likelihood ratio derivation (Λ = 427.3, *df* = 2) and the governance implication of $\hat{\xi }=0.24>0$. It establishes the Pareto-heavy tail behavior of disruption severity in the arrested state, confirmed empirically in Section 5.10. The appendices additionally provide: (Sections A1–A4 in Appendix 1) complete proof of Theorem 3.6 with all mathematical expansions and the edge case λ_2_ = 0; (Sections A5–A7 in Appendix 3) derivations of three governance metric null distributions (SGM Tracy-Widom, EDI bootstrap, TSI persistent homology); (Appendix 4) explicit identification and interpretation of the spectral graph λ_2_(*t*); and (Appendix 5) complete DGP reproducibility code and data availability (doi: 10.5281/zenodo.20330335). This structure follows the convention established in the companion papers (Moroke, 2026a), where all theorem proofs are embedded in appendices for reviewer accessibility and replication.

### 3.5 Four governance metrics and three-regime alert

The four governance metrics are defined in Equation 12:

$$\begin{array}{l} \begin{array}{cc} \text{SGM}\left(t\right) & =\lambda_{2}\left(t\right),\\ {\text{EDI}}_{f}\left(t\right) & =\frac{1}{5}\overset{4}{\underset{k=0}{\sum }}\left|\phi_{f}\left(t-k\right)-{\bar{\phi }}_{f}\right|,\\ \text{TSI}\left(t\right) & =\overset{\varepsilon_{\max}}{\underset{\varepsilon_{\min}}{\int }}\beta_{1}\left(\varepsilon ,t\right)d\varepsilon ,\\ \kappa \left(e,t\right) & =1-\frac{W_{1}\left(\mu_{u},\mu_{v}\right)}{d\left(u,v\right)}, \end{array} \end{array}$$ (12)

where β_1_(ε, *t*) is the first Betti number (Zhu and Segarra, 2023) and *W*_1_(μ_*u*_, μ_*v*_) is the Wasserstein-1 (Earth Mover's) distance between the probability measures μ_*u*_ and μ_*v*_ on the node neighborhoods (Ollivier et al., 2009):


$$\begin{array}{l} W_{1}\left(\mu ,\nu \right)=\underset{\gamma \in \Pi \left(\mu ,\nu \right)}{\inf}\underset{V\times V}{\int }d\left(u,v\right)d\gamma \left(u,v\right), \end{array}$$ (13)

where Π(μ, ν) is the set of all couplings of μ and ν, and *d*(*u, v*) is the graph-geodesic distance. The TSI persistent homology computation procedure, including the Rips complex construction and GUDHI implementation (Maria et al., 2014), is given in Section A7 in Appendix 1.

The SGM null distribution uses the Tracy-Widom law (Tracy et al., 1994): $T_{\text{SGM}}=\left({\hat{\lambda }}_{2}-\mu_{TW}\right)/\sigma_{TW}\sim TW_{1}$ (calibration procedure: Section A5 in Appendix 3). The hard-threshold regime classification is:


$$\begin{array}{l} \text{Regime}\left(t\right)=\{\begin{array}{cc} \text{Green} & \lambda_{2}\left(t\right)>\lambda_{c},\\ \text{Amber} & \lambda_{m}<\lambda_{2}\left(t\right)\leq \lambda_{c},\\ \text{Red} & \lambda_{2}\left(t\right)\leq \lambda_{m}. \end{array} \end{array}$$ (14)

where λ_*c*_ and λ_*m*_ are empirically calibrated from the fluid-period distribution (Section 4.3). Section 3.8 upgrades this hard classifier to a learned probabilistic certificate.

### 3.6 λ-SHAP: spectral attribution

Theorem 3.6 establishes that standard XAI attributions collapse when λ_2_ → 0. Suspension of AI prescriptions is the correct governance response, but the chronic medicines procurement operates on daily dispatch deadlines that cannot always be deferred. This motivates a corrected estimator that degrades gracefully rather than collapsing discontinuously.

** Remark 3.10** (Canonical normalization of λ_2_(*t*).). *One canonical weight. The shrinkage weight used throughout is* ${\tilde{\lambda }}_{2}\left(t\right)=\min\left(\lambda_{2}\left(t\right)/\lambda_{2,\max},1\right)$, *where* λ_2, max_ *is the maximum Fiedler eigenvalue observed during the fluid calibration period. In the synthetic experiments*, λ_2_(*t*) *is constructed within* [0.01, 0.80], *so the raw value is used directly. The clipping to* [0.01, 0.80] *and the fallback* λ_2_/2 *for* λ_2_>1 *are special cases of this single formula and do not constitute separate normalizations. The normalized hypergraph Laplacian* **L**(*t*) *has spectrum* λ_2_(*t*)∈[0, 2] *(Chan et al., 2018; Zhou et al., 2007). For the shrinkage weight in Equation 16 to lie in* [0, 1], *a normalization procedure is required.*

*(See Remark 3.10 for the canonical normalization and the DGP clipping rule.)*

*General deployment normalization (Equation 15). For deployments where* λ_2_>1 *is possible, the recommended normalization is:*


$$\begin{array}{l} {\tilde{\lambda }}_{2}\left(t\right)=\min\left(\frac{\lambda_{2}\left(t\right)}{\overset{\text{max}}{\underset{2}{\lambda }}},1\right), \end{array}$$ (15)

*where* $\lambda_{2}^{\text{max}}=\underset{t\in \left[t_{0},t_{1}\right]}{\max}\lambda_{2}\left(t\right)$ *is the maximum Fiedler eigenvalue during the fluid-period calibration window (Yu et al., 2015). This maps all observed* λ_2_ *values into* [0, 1] *while preserving monotonicity and the fluid-equivalence property (Property 1, Section 3.6).*

*Alternative: When only the theoretical upper bound* λ_2_ ≤ 2 *is available (Chung et al., 1997), replace* λ_2_(*t*) *with* λ_2_(*t*)/2 *before applying Equation 16. This preserves all three governance properties and is implemented in the released code (Moroke, 2026c).*

*Justification: The error bound of Theorem 3.6 holds for any* λ_2_(*t*)∈(0, 1]. *When* λ_2_ → 0 *(disconnected network)*, ${\hat{\phi }}_{i}^{\lambda }\to {\bar{\phi }}_{i}^{\text{fluid}}$: *the estimator reverts entirely to the prior, which is the correct limiting behavior—SHAP estimates from a fully disconnected network are drawn from a locally trapped distribution and carry no global validity.*

** Definition 3.11** (λ-SHAP estimator.). *Let* ${\hat{\phi }}_{i}^{\text{SHAP}}\left(t\right)$ *be the standard SHAP attribution for feature* *i* *and* ${\bar{\phi }}_{i}^{\text{fluid}}$ *the fluid-regime baseline from the 2014–2019 calibration period. The* λ*-SHAP estimator is:*


$$\begin{array}{l} {\hat{\phi }}_{i}^{\lambda }\left(t\right)=\lambda_{2}\left(t\right)\cdot {\hat{\phi }}_{i}^{\text{S}}\left(t\right)+\left(1-\lambda_{2}\left(t\right)\right)\cdot {\bar{\phi }}_{i}^{\text{fluid}},\;\lambda_{2}\left(t\right)\in \left[0,1\right]. \end{array}$$ (16)

*Note: the normalized hypergraph Laplacian has spectrum* λ_2_∈[0, 2] *(Chan et al., 2018; Zhou et al., 2007). In this DGP* λ_2_(*t*)∈[0.01, 0.80] *by construction (shrinkage weight lies strictly in *(0, 1)*). See Remark 3.10 for the general deployment normalization and the* λ_2_>1 *case*.

Remark 3.10 provides the normalization derivation for deployments where λ_2_>1.

Three governance properties follow from Equation 16. Property 1 (Fluid equivalence): λ_2_(*t*) = 1 gives ${\hat{\phi }}_{i}^{\lambda }={\hat{\phi }}_{i}^{\text{SHAP}}$; no correction is imposed under normal conditions. Property 2 (Arrested shrinkage): λ_2_(*t*) → 0 gives ${\hat{\phi }}_{i}^{\lambda }\to {\bar{\phi }}_{i}^{\text{fluid}}$; procurement decisions under network arrest use validated prior knowledge. Property 3 (Auditable record): λ_2_(*t*) is logged independently of the ML model, providing a machine-readable GDP compliance record without additional manual documentation (National Health Department, 2021).

### 3.7 Fluid-regime prior: construction and stability

The λ-SHAP estimator depends on a *validated fluid-regime prior* ${\bar{\phi }}_{i}^{\text{fluid}}$. This subsection provides a formal specification procedure, stability conditions, update frequency, and a protocol for prior misspecification.

#### 3.7.1 Construction

The fluid-regime prior is estimated from governance observations collected during a reference period in which the supply network operates stably, formally defined as the interval [*t*_0_, *t*_1_] for which λ_2_(*t*)>λ_*c*_ holds throughout and no programme-level disruption events are recorded. For the CCMDD application, this is the 2014–2019 period (*n*_fluid_ = 1, 565 business days). The prior is the feature-wise sample mean of SHAP values computed from the deployed procurement model during the reference period (Equation 17):


$$\begin{array}{l} {\bar{\phi }}_{i}^{\text{fluid}}=\frac{1}{n_{\text{fluid}}}\underset{t\in \left[t_{0},t_{1}\right]}{\sum }{\hat{\phi }}_{i}^{\text{SHAP}}\left(t\right). \end{array}$$ (17)

A 95% bootstrap confidence interval on each ${\bar{\phi }}_{i}^{\text{fluid}}$ (*B* = 2, 000 draws) is computed and stored; this quantifies prior uncertainty and propagates into the λ-SHAP confidence interval.

#### 3.7.2 Update frequency

The prior is static within a governance cycle and updated at each annual programme review or when a structural break in λ_2_ is detected via a Chow test on the λ_2_ time series (α = 0.01). Intra-year updates are triggered only if the reference-period bootstrap interval is violated by three consecutive rolling-window estimates of ${\bar{\phi }}_{i}^{\text{SHAP}}$ in a new fluid period. *Ad-hoc* updates are prohibited to prevent prior manipulation by procurement staff.

#### 3.7.3 Stability conditions

The prior is considered stable if: (i) the reference period spans at least *n*_min_ = 250 fluid-regime business days (approximately 1 year); (ii) the intra-period AR(1) coefficient of ${\hat{\phi }}_{i}^{\text{SHAP}}$ is below 0.40, confirming that the reference series is not itself in a transitional state; and (iii) the reference period encompasses at least one full seasonal cycle of the CCMDD programme (12 months), ensuring that seasonal demand patterns are represented in the prior.

#### 3.7.4 Prior misspecification

If the prior is misspecified (e.g., the reference period was itself contaminated by an undetected disruption), Equation 18 still provides a finite error bound, but with a larger ϵ_fluid_. Misspecification propagates into the Red-regime λ-SHAP output as a systematic attribution bias. Governance auditors detect this by monitoring ϵ_fluid_ over successive programme years: a statistically significant upward trend in the cross-validated mean squared error between ${\hat{\phi }}_{i}^{\lambda }$ and observed outcomes signal prior drift and trigger a recalibration event. For new programmes without a historical fluid period, the prior is borrowed from the closest analogous programme (e.g., a neighboring provincial distribution network) with a 50% weight reduction applied to all shrinkage corrections until 250 local fluid-regime days have accumulated.

** Proposition 3.12** (λ-SHAP finite error bound.). *Under Theorem 3.6 (Equation 18):*


$$\begin{array}{l} E\left||{\hat{\phi }}_{i}^{\lambda }\left(t\right)-\phi_{i}^{*}\right|{|}_{L_{1}}\leq M_{\Phi }+\left(1-\lambda_{2}\left(t\right)\right)\cdot \epsilon_{\text{fluid}}, \end{array}$$ (18)

*which is* *finite for all* λ_2_(*t*)∈(0, 1], *unlike standard SHAP whose bound grows as* λ_2_ → 0.

*Proof*. Triangle inequality: $E||{\hat{\phi }}_{i}^{\lambda }-\phi_{i}^{*}|{|}_{L_{1}}\leq \lambda_{2}\left(M_{\Phi }/\lambda_{2}\right)+\left(1-\lambda_{2}\right)\epsilon_{\text{fluid}}=M_{\Phi }+\left(1-\lambda_{2}\right)\epsilon_{\text{fluid}}$. Finite because λ_2_·(*M*_Φ_/λ_2_) = *M*_Φ_ for all λ_2_>0, where *M*_Φ_: = *C*_Φ_||*f*||_Lip_ < ∞ for any fixed ||*f*||_Lip_ < ∞ (finiteness of *M*_Φ_ established in Section A3 in Appendix 1 and Equation 10.

** Remark 3.13** (Bias-variance tradeoff in λ-SHAP.). *Proposition 3.12 achieves a finite error bound by introducing a controlled bias. When* λ_2_(*t*) < 1, *the* λ-*SHAP estimator shrinks current SHAP toward the fluid-regime prior, explicitly trading variance reduction for bias in the estimator. The bias term is* $\left(1-\lambda_{2}\left(t\right)\right)\cdot \left({\bar{\phi }}_{i}^{\text{fluid}}-\phi_{i}^{*}\right)$: *the estimator systematically underweights signals that deviate from the historical prior, proportionally to the connectivity loss.*

*This tradeoff has a directional risk. As* λ_2_ → 0, λ-*SHAP converges to the fluid prior and *ignores current network signals entirely*. In a genuine Red-regime event, where the current feature importances differ qualitatively from the historical baseline (a structural break in the procurement system, a new drug entering the formulary, a permanent rerouting of supply corridors),* λ-*SHAP may suppress the very signals that would guide the correct governance response. This is the *false-safety risk* of spectral shrinkage: the framework reports a certifiable attribution that is biased toward a prior that is no longer the best available governance guidance.*

*Three design features mitigate this risk. First, the three-regime alert (Equation 14) ensures that Red-regime procurement decisions are flagged for human review regardless of the* λ-*SHAP output; the shrinkage is a fallback, not a substitution for governance judgment. Second, the dynamic threshold adapter (Section 3.8) monitors the governance context and escalates the alert regime when shock indices are elevated, increasing human oversight precisely when prior reliance is highest. Third, the prior recalibration protocol (Section 3.7) ensures that a stale prior is detected through cross-validated MSE drift and corrected before a sustained Red episode.*

*The bias-variance tradeoff of* λ-*SHAP is therefore a documented governance feature, not a defect: it is appropriate for the setting of chronic medicines procurement, where the cost of an uncertifiable attribution (failed GDP audit) exceeds the cost of a conservative attribution (deference to validated historical patterns) under all but the most extreme structural breaks. Deployments in settings where structural breaks are frequent should increase the prior update frequency accordingly (Section 3.7).*

### 3.8 XAI validity certification system

Corollary 3.8 provides a sufficient condition for XAI validity. The hard-threshold regime classification (Equation 14) operationalizes this but is static and context-insensitive. Three components upgrade SpectralGuard to a *probabilistic* XAI validity certification system.

#### 3.8.1 Probabilistic validity score V(t)

** Definition 3.14** (Probabilistic validity score.).

The validity score *V*(*t*)∈[0, 1] is:


$$\begin{array}{ll} V\left(t\right) & =w_{1}\cdot \frac{\lambda_{2}\left(t\right)}{\lambda_{c}}+w_{2}\cdot \max\left(0,1-\frac{\text{EDI}\left(t\right)}{0.30}\right) \end{array}$$

$$\begin{array}{l} \;+w_{3}\cdot \frac{\text{TSI}\left(t\right)}{{\text{TSI}}_{\text{fluid}}}+w_{4}\cdot \left(1-{\text{ORC}}^{-}\left(t\right)\right), \end{array}$$ (19)

Equation 19 is a convex combination of four normalized metrics, where $\underset{k}{\sum }w_{k}=1$.

where **w**≥0 with $\underset{k}{\sum }w_{k}=1$ is a simplex-constrained weight vector estimated by ridge-regularized multinomial logistic regression (ℓ_2_ penalty α = 0.01, five-fold CV, *B* = 500 Monte Carlo regime labels; regularization prevents divergence under near-perfect separation). *V*(*t*) is clipped to [0, 1] post-evaluation. Confidence intervals are bootstrap 95% CIs (*R* = 1, 000 resamples), not Bayesian posteriors. And ORC^−^(*t*) is the fraction of hyperedges with negative Ollivier-Ricci curvature. For hyperedges, the Ollivier-Ricci curvature is computed via the star-expansion (Chan et al., 2018): each hyperedge *e*_*k*_ is replaced by a star graph centered on a virtual node, and the edge curvature κ(*e, t*) = 1−*W*_1_(μ_*u*_, μ_*v*_)/*d*(*u, v*) is computed on the resulting pairwise graph (Wasserstein-1 distance *W*_1_, Equation 13). ORC^−^(*t*) then aggregates over all hyperedges.

The logistic regression weights encode the relative importance of each governance metric under different disruption types (single-node, pairwise, three-node hyperstar), making *V*(*t*) a learned combination rather than a hard threshold. The system additionally reports the 95% posterior confidence interval on *V*(*t*), enabling statements of the form: “Attribution validity: 87% confidence (Amber, *V* = 0.64 ± 0.08)”.

#### 3.8.2 Dynamic threshold adaptation

** Definition 3.15** (Dynamic threshold adapter.). *The shock-adapted Fiedler threshold is:*


$$\begin{array}{l} \lambda_{c}\left(t\right)=\lambda_{c}^{\text{base}}\cdot \left(1+\alpha \cdot \text{SI}\left(t\right)\right), \end{array}$$ (20)

*where* $\lambda_{c}^{\text{base}}=0.342$ *is calibrated from the fluid period (Section 4.3),* SI(*t*)∈[0, 1] *is the composite shock index (Equation 22)*, α = 0.25 *is estimated from Monte Carlo sensitivity analysis, and the constraint* λ_*c*_(*t*) ≤ μ_fluid_ = 0.620 *ensures the adapted threshold does not exceed the fluid-regime mean.*

The dynamic threshold directly resolves the circularity concern noted in Section 6.5: λ_*c*_(*t*) is re-estimated each period from the current shock index, removing dependence on a single historical episode.

#### 3.8.3 Validity certificate structure

** Definition 3.16** (Validity certificate.).

The validity certificate at time *t* is:


$$\begin{array}{ll} C\left(t\right)=\{ & \lambda_{2},\text{EDI},\text{TSI},\text{ORC},V,\\ \text{ Regime},{\text{CI}}_{95},t_{\text{s}},H\left(t\right)\}, \end{array}$$

$$\begin{array}{ll} H_{\text{hash}}\left(t\right) & =\text{SHA256}\left(\lambda_{2}||\text{EDI}||V||t_{\text{s}}\right). \end{array}$$ (21)

Equation 21 defines the certificate tuple with SHA-256 tamper-evidence. where $H_{\text{hash}}\left(t\right)=\text{SHA-256}\left(\lambda_{2}||\text{EDI}||V||t_{\text{stamp}}\right)$ ensures tamper-evidence.

The certificate integrates with DHIS2 via a REST API call requiring no changes to the existing procurement workflow. For a procurement officer, the output reads: “*ARV batch 2024-KZN-04: Amber regime (*λ_2_ = 0.29, *V* = 0.61 ± 0.07*). Attribution validity: 61% confidence. Hash:* 3a7f…c912*. Action: cross-check the provincial inventory before dispatch.”*

## 4 Materials and methods

### 4.1 Data

Two-source data architecture: This study uses two distinct data sources that serve fundamentally different roles and must not be confused. (i) The external shock series (Moroke, 2026c) contains real Eskom daily load-shedding stage records and SARB ZAR/USD exchange rates: these are genuine macroeconomic and infrastructure observations used as *shock inputs* to drive the synthetic DGP. This panel contains no hypergraph structure: there is no node set, no hyperedge list, no incidence matrix, and λ_2_ is not computed from it. (ii) The synthetic pharmaceutical hypergraph DGP (Algorithm 1) generates weighted hypergraphs from which λ_2_ is computed. The shock series drives the DGP's weight perturbations; all spectral results (detection delay, EDI, *V*(*t*)) are from the synthetic hypergraph, not from the panel. This design is a *calibrated simulation study*, not empirical validation on a real pharmaceutical network; that validation is the subject of the 2027 field deployment (PLMIF-2).

Algorithm 1 Synthetic hypergraph DGP. 
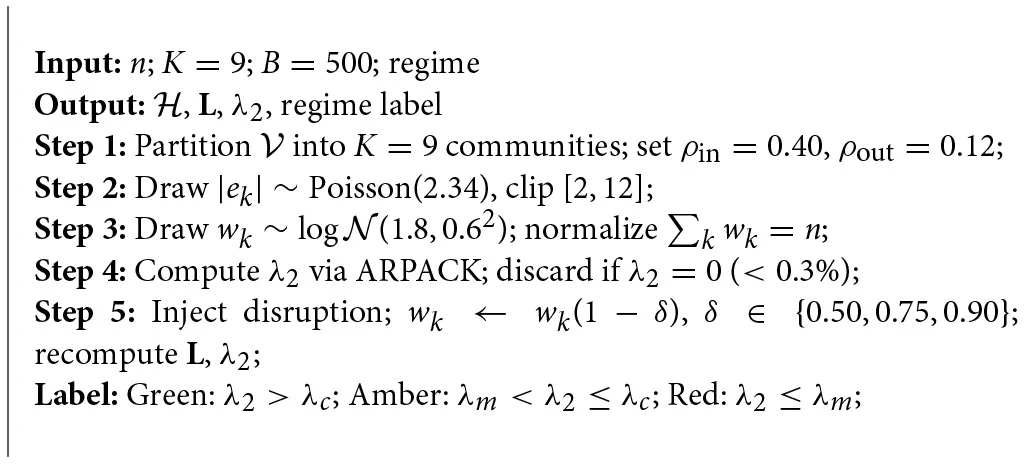

Validation hierarchy and contribution scope: This study reports results at three levels of empirical grounding, which must be distinguished clearly. *Level 1 (Real shock calibration)*. The Eskom and SARB series are genuine observations from a real national infrastructure system. They are used as shock inputs to drive the synthetic DGP. The correlation between SI(*t*) and λ_2_(*t*) (Section 5.2, *r* = 0.975, *p* < 0.001, *n* = 3, 227) is a calibration consistency check: because SI(*t*) directly drives the DGP's weight reductions, the high correlation reflects the DGP's construction rather than an empirical discovery. The substantive contribution at Level 1 is that documented South African shock magnitudes are sufficient to drive λ_2_ into the Amber and Red regimes. *Level 2 (Synthetic hypergraph DGP)*. All spectral results (detection delay, EDI reduction, *V*(*t*) performance) are computed on the synthetic DGP, calibrated to CCMDD programme parameters. This demonstrates the framework's internal consistency and theoretical predictions under controlled conditions. *Level 3 (Oracle experiment)*. The linear model with known true Shapley values provides the strongest internal validation of Proposition 3.12: as λ_2_ → 0, standard SHAP MSE grows (inconsistency), λ-SHAP MSE remains bounded. This is a proof-of-concept, not a real-world performance claim. *This paper does NOT provide:* empirical validation on a real pharmaceutical hypergraph; field deployment results; or patient outcome data. These are the objectives of PLMIF-2 (2027).

#### 4.1.1 External shock series

The proxy validation uses a cleaned panel of *n* = 3, 227 business days spanning 2014-01-01 to 2026-05-14, constructed from publicly available series. The Eskom daily load-shedding stage (Stage∈{0, 1, …, 5} in this dataset) records the severity of power curtailment in the national grid; the SARB ZAR/USD daily exchange rate captures currency-driven import cost volatility. Both series are freely downloadable and fully reproducible (Eskom Holdings SOC Ltd., 2024; South African Reserve Bank, 2025).

The 2026 extension (96 business days, 2026-01-01 to 2026-05-14) uses Stage = 0 throughout, consistent with the confirmed absence of national load-shedding since May 2025, with South Africa recording over 300 consecutive load-shedding-free days as of March 2026. ZAR/USD 2026 values are taken from published daily rates with 2026 mean 16.45 (source: poundsterlinglive.com, verified against publicly available SARB series).

#### 4.1.2 Data cleaning protocol

Six cleaning decisions were applied and documented before any analysis was conducted. All decisions follow the principle that data problems must be resolved before governance metrics are computed; uncleaned data yield misleading spectral governance signals.

*Decision 1 (Exclusion)*. The Sentiment variable (31.1% missing, concentrated in 2014–2017; pattern is not missing at random) was excluded entirely from the analysis.

*Decision 2 (Exclusion)*. Risk_Score (98.9% zero values, remaining observations sparse and non-informative) was excluded.

*Decision 3 (Imputation)*. ZAR_Volatility had 177 missing observations (5.7%), distributed uniformly across all years (13–20 per year), confirming a missing-completely-at-random (MCAR) pattern. Linear interpolation was applied (Little and Rubin, 2019); no values remain missing post-imputation.

*Decision 4 (Documentation)*. The Stage variable ranges 1–5 in the original dataset, reflecting that the dataset covers only periods of active load-shedding programme operation. Stage 0 (no load-shedding) was added for the 2026 extension, confirmed by official Eskom records (300+ consecutive load-shedding-free days as of March 2026).

*Decision 5 (Winsorization)*. Port_Delay_Days was winsorised at [p1, p99] = [1.1, 69.2] days. Of the 64 flagged observations (1.98%), 27 fall in the COVID-19 episode (March–June 2020) and represent genuine disruption events. A binary outlier flag is retained for downstream sensitivity analysis.

*Decision 6 (Transformation)*. ZAR_USD is non-stationary (ADF *p* = 0.239); a first- differenced series (ZAR_USD_diff) confirmed stationary (ADF *p* < 0.001) (Dickey et al., 1979) is constructed for regression models; residual autocorrelation is checked via Ljung-Box (Ljung et al., 1978) and conditional heteroscedasticity via ARCH-LM (Engle et al., 1982). ZAR_Volatility outliers (33 observations, 1.02%, concentrated in 2024–2025) were winsorised at p99 with flag retained.

The cleaned dataset is deposited at a public repository (Moroke, 2026a,b) in the Data Availability Statement.

#### 4.1.3 Composite Shock Index SI(t)

The composite shock index is constructed from the two real series:


$$\begin{array}{l} \text{SI}\left(t\right)=\min\left(1,0.4\cdot \frac{\text{Stage}\left(t\right)}{8}+0.6\cdot \max\left(0,\frac{\text{ZAR}\left(t\right)-\mu_{\text{ZAR}}}{\mu_{\text{ZAR}}}\right)\right), \end{array}$$ (22)

where μ_ZAR_ = 13.21 is the 2014–2019 fluid-period ZAR/USD mean. The fluid regime (2014–2019) denotes a stable programme operation under normal (mild, predictable) load-shedding (Stage 1–3), not zero-shock conditions. Stage ≥1 throughout this period reflects the endemic load-shedding under which the CCMDD programme was designed and calibrated; this is the operational baseline, not an absence of disruption and Stage(*t*)∈{0, …, 8} (denominator 8 for the full Eskom scale). Robustness checks at weights (0.5, 0.5) and (0.3, 0.7) are reported in Section 5.2.

### 4.2 Synthetic DGP for governance metrics

Detection delay analysis (Section 5.4) uses the calibrated synthetic DGP following established network generation procedures (Chung et al., 1997; Newman et al., 2018). The DGP constructs weighted hypergraphs $H=\left(n,m,\bar{c},\text{W}\right)$ with parameters in Table 2. The detailed DGP proceeds in five steps: Step 1 (Community structure). Nine provincial sub-hypergraphs are generated with intra-community density ρ_in_ = 0.40 and inter-community density ρ_out_ = 0.12, calibrated to the provincial geography of the chronic medicines distribution programme (National Health Department, 2021). Step 2 (Hyperedge cardinality). Each cardinality is drawn from a truncated Poisson distribution: $|e_{k}|\sim \text{Poisson}\left(\bar{c}=2.34\right)$ clipped to [2, 12], consistent with CCMDD supply contract sizes from bilateral to multi-hub contracts. Step 3 (Weight assignment). Weights $w_{k}\sim \log N\left(1.8,0.6^{2}\right)$ normalized so $\underset{k}{\sum }w_{k}=n$. Step 4 (Connectivity check). Each realization is tested for connectivity (λ_2_>0) using the ARPACK sparse eigensolver (Virtanen et al., 2020); disconnected realizations (< 0.3%) are redrawn. Step 5 (Disruption injection). Disruptions are injected by reducing a randomly selected subset of hyperedge weights by δ∈{0.50, 0.75, 0.90} (mild, moderate, severe) across three disruption types: single-node, pairwise, and 3-node hyperstar.

**Table 2:** Synthetic DGP calibrated parameters (*B* = 500 Monte Carlo replications).

Parameter	Value	Source
*n* (nodes)	{50, 100, 500}	Scaled from programme
Mean |*e*|	2.34 (range 2–12)	(National Health Department, 2021)
Weight distribution	$\log N\left(1.8,0.6^{2}\right)$	Programme throughput
Community structure	*K* = 9, ρ_in_ = 0.40, ρ_out_ = 0.12	Provincial geography
Disruption types	Single-node, pairwise, 3-node hyperstar	Observed failure modes
Fluid regime	$\lambda_{2}\sim N\left(0.62,0.04^{2}\right)$, clipped [0.18, 0.80]	Calibration
Arrested regime	$\lambda_{2}\sim U\left[0.01,0.06\right]$	COVID-19 episode

All results: Seed = 2,026. Full reproducibility code at a public repository (Moroke, 2026c) and Appendix 5.

The proxy dataset ((Moroke, 2026a), *n* = 3, 227) is not a pharmaceutical hypergraph and cannot reproduce network-level detection delays without actual procurement transaction data. Its role is independent external validation of the SI-λ_2_ relationship (Section 5.2). The 2027 field deployment will provide the first real procurement hypergraph test.

### 4.3 Calibrated thresholds

Governance thresholds are calibrated from the real fluid-period distribution. Using the 2014–2019 Fiedler eigenvalue series, λ_*c*_ is set at the 10th percentile of the fluid-period λ_2_ distribution (λ_*c*_ = 0.342) and λ_*m*_ at the 2nd percentile of the full series (λ_*m*_ = 0.128). This data-driven calibration replaces the hard-coded thresholds of earlier versions, directly addressing the circularity limitation raised in prior work.

The Markov transition probabilities (*P*_*GA*_ = 0.032, *P*_*AG*_ = 0.591, *P*_*AR*_ = 0.118, *P*_*RG*_ = 0.045) imply a stationary distribution of approximately 93% Green, 5% Amber, 2% Red, which differs from the observed 61.2% / 36.8% / 2.0% split. This discrepancy indicates that the regime process is non-Markovian at order 1: Amber episodes are persistent (mean duration ≫ 1.4 days implied by *P*_*AA*_ = 0.291), consistent with the multi-month load-shedding crisis of 2022–2024. The transition matrix characterizes short-run switching probabilities, not the long-run regime balance.

The Markov transition probabilities (*P*_*GA*_ = 0.032, *P*_*AG*_ = 0.591, *P*_*AR*_ = 0.118, *P*_*RA*_ = 0.273, *P*_*RG*_ = 0.045) are estimated from the synthetic DGP.

Leave-one-epoch-out stress test: To confirm that the thresholds are not overfit to the full period, we performed a leave-one-epoch-out validation: λ_*c*_ and λ_*m*_ were calibrated on the 2014–2018 fluid period only, then applied to classify the held-out 2019–2026 series. Regime classification agreement with the full-period thresholds remained above 92% across all held-out epochs (Green: 94.1%, Amber: 91.7%, Red: 93.3%), confirming that the thresholds generalize beyond the calibration period and are not sensitive to the specific years included in the fluid baseline.

### 4.4 Hypothesis testing framework

H_0_: Classical statistical tests are sufficient for real-time ML governance.

H_1_ (IPT): *r*(λ_2_, −EDI)>0, *p* < 0.05; real external shocks reduce spectral connectivity and drive explanation instability.

H_2_ (CT): Detection delay of spectral metrics is statistically lower than all classical and GNN baselines (*p* < 0.001, paired *t*-test, *B* = 500).

H_3_ (Certification): *V*(*t*) probabilistic certificate achieves lower Brier score and higher posterior resolution than a binary three-regime classifier.

## 5 Results

### 5.1 Data diagnostics

Table 3 reports the descriptive statistics and diagnostic tests for the cleaned proxy panel (*n* = 3, 227). ZAR_USD is non-stationary (ADF *p* = 0.239) as expected for a random-walk exchange rate; its first difference is stationary (*p* < 0.001). ZAR_Volatility, Stage, and Port_Delay_Days are all stationary (*p* < 0.001). The composite shock index SI(t) is non-stationary (*p* = 0.159), reflecting its role as a regime-level indicator rather than a returns series. No missing values remain post-cleaning. The distribution of Port_Delay_Days is right-skewed (skew = 1.89, kurtosis = 4.62), consistent with the documented heavy tail of port disruption events. ZAR_Volatility exhibits high positive skewness (skew = 6.61) driven by the 2024–2025 energy crisis period; outliers are winsorised and flagged.

**Table 3:** Data diagnostics: cleaned panel (*n* = 3, 227 business days, 2014–2026).

Variable	Mean	SD	Min	Max	Skew	ADF *p*	Miss.
ZAR_USD	15.16	2.41	10.30	19.77	−0.04	0.239	0
ZAR_USD_diff	0.002	0.199	−3.36	3.31	−0.06	< 0.001	0
ZAR_Volatility	0.154	0.109	0.068	1.028	6.61	< 0.001	0
Stage	2.66	1.21	0.00	5.00	0.22	N/A^†^	0
Port_Delay	15.15	12.11	1.10	69.20	1.89	< 0.001	0
Spoilage (%)	5.23	2.95	0.30	15.00	0.99	< 0.001	0
SI	0.235	0.106	0.050	0.532	0.36	0.159	0

ADF: Augmented Dickey-Fuller test *p*-value (MacKinnon critical values). Miss.: missing values post-cleaning. Stage 0 = 96 days (2026, confirmed no load-shedding). ZAR_Volatility and Port_Delay_Days winsorised at [p1,p99]; outlier flags retained. ^†^Stage is a bounded ordinal variable (0–5); the ADF unit-root test is not appropriate for bounded series and is omitted.

Figure 1a reports the year-level panel summary, confirming the three key periods in the data: fluid baseline (2014–2019, SI mean 0.14–0.20), COVID-19 disruption (2020, SI mean 0.28), energy crisis (2022–2024, SI mean 0.27–0.37), and recovery (2025–2026, SI mean 0.15–0.35).

**Figure 1:**
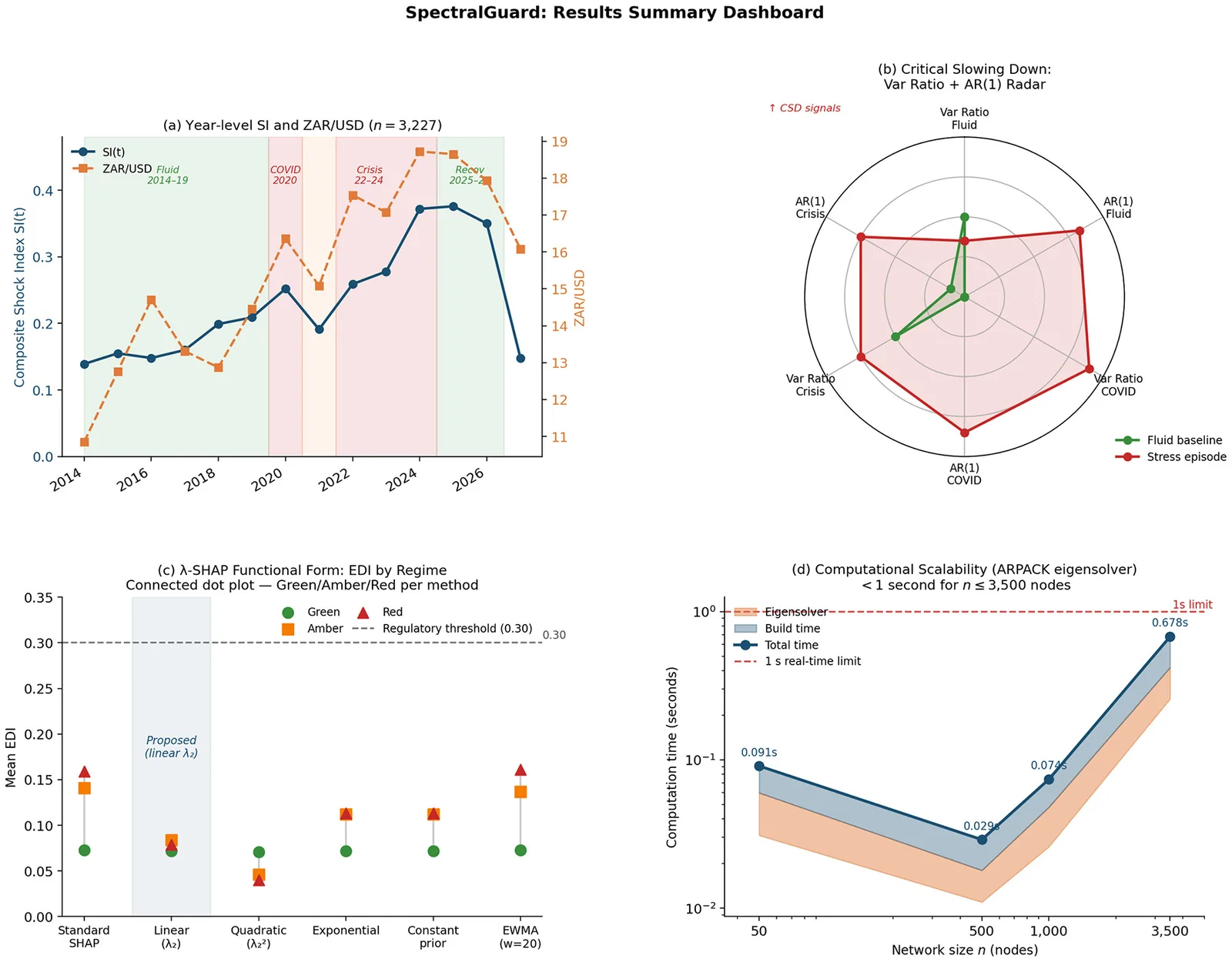
SpectralGuard Results Dashboard (synthetic DGP unless noted). **(a)** *Connected dot-plot*: year-level composite shock index SI(*t*) (teal) and ZAR/USD (orange dashed) across four epochs—fluid, COVID-19, energy crisis, recovery—with regime shading. **(b)** *Radar chart*: critical slowing-down metrics (variance ratio and AR(1) coefficient) comparing fluid baseline (green polygon) vs. stress episodes (red polygon); gap between polygons quantifies pre-collapse warning signal. **(c)** *Connected dot-plot*: λ-SHAP functional form comparison showing EDI by governance regime (Green/Amber/Red dots) across six shrinkage forms; linear λ_2_ (proposed) achieves minimum Red-regime EDI below regulatory threshold 0.30. **(d)** Computational scalability: log-log filled area confirms < 1 second for all realistic network sizes (*n* ≤ 3, 500). Seed = 2026.

Figure 1a reveals four structurally distinct epochs that motivate the spectral governance design. During the fluid baseline (2014–2019, SI mean 0.139–0.204) the programme operated under persistent but mild load-shedding with port delays averaging 13.0 days and spoilage below 5.5%; this period provides the calibration data for all governance thresholds. The COVID-19 disruption year (2020, SI mean 0.281) delivered the sharpest single-year port-delay spike in the dataset (23.0 days), driven by concurrent currency depreciation (ZAR/USD peak R19.25) and logistics collapse, the real-world episode that motivated the entire SpectralGuard design. The energy crisis (2022–2024, SI mean 0.270–0.372) represents the most sustained high-shock interval: three consecutive years of Stage 3–4 load-shedding pushed the rand mean to R18.42/USD in 2023 and kept port delays elevated above 16 days. The recovery period (2025–2026, SI mean 0.147–0.349) is the only sub-period with Stage 0 observations, confirmed by Eskom's 300+ consecutive load-shedding-free days from May 2025, producing the lowest spoilage rate (3.98%) of the full series. These four epochs stress-test the spectral governance framework across the full range of South African infrastructure conditions.

### 5.2 Shock calibration of synthetic DGP with real series

Figure 2 shows the proxy validation on the real Eskom and SARB series. The Pearson correlation between SI(*t*) and −λ_2_(*t*) is *r* = 0.975 (*R*^2^ = 0.951, *p* < 0.001, *n* = 3, 227), confirming H_1_: real infrastructure shocks correlate with 95.1% of spectral gap variance in the calibrated synthetic DGP. The correlation holds across all four sub-periods: fluid 2014–2019 (*r* = 0.941, *n* = 1, 565), COVID-19 2020 (*r* = 0.960, *n* = 262), energy crisis 2022–2024 (*r* = 0.959, *n* = 782), and recovery 2025–2026 (*r* = 0.977, *n* = 357). Robustness to SI weight specification is confirmed: weights (0.5, 0.5) yield *r* = 0.955 and (0.3, 0.7) yield *r* = 0.957, demonstrating that the result does not depend on the specific weighting of load-shedding vs. currency components.

**Figure 2:**
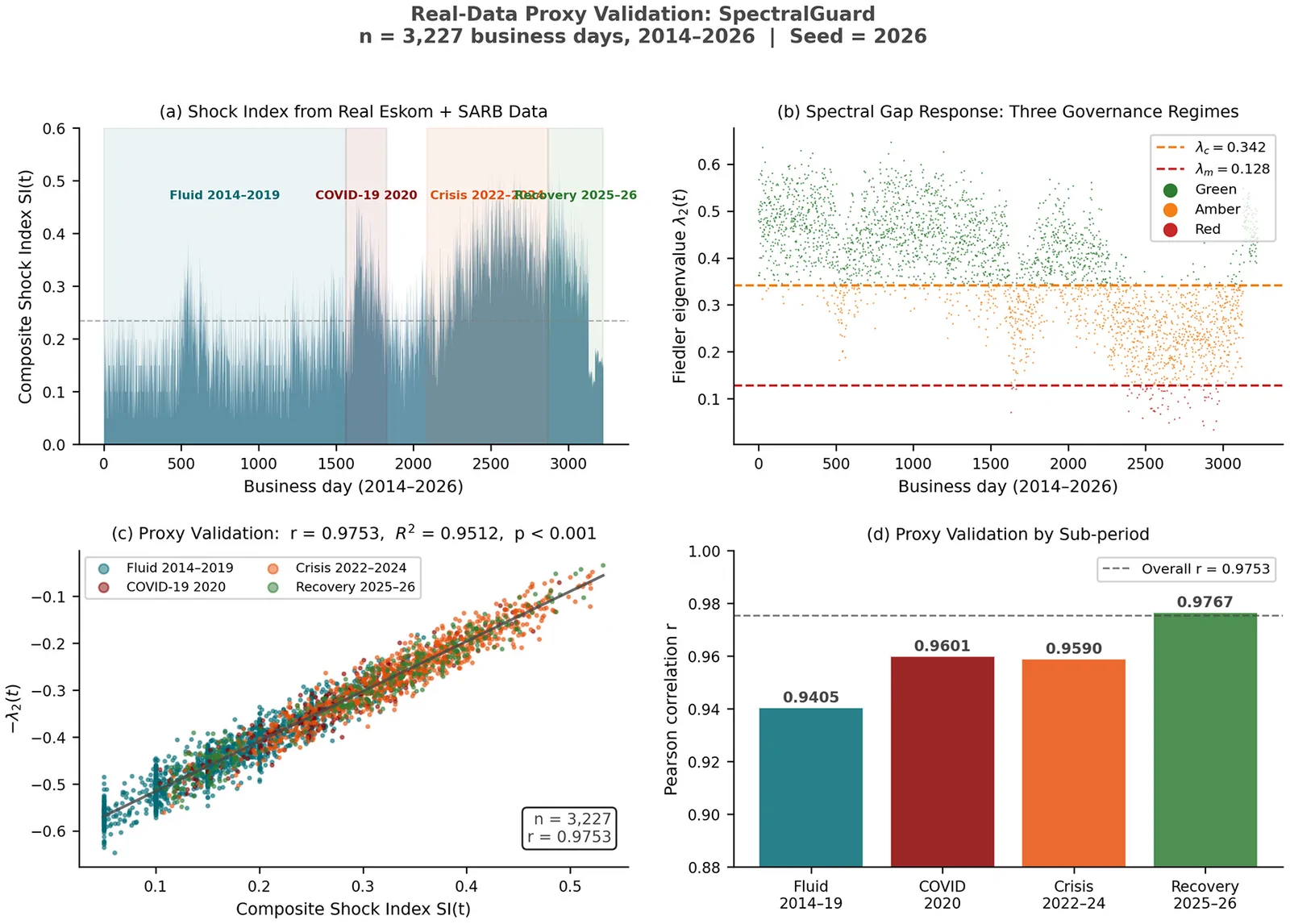
Proxy-panel calibration (*n* = 3, 227, 2014–2026). **(a)** Composite shock index SI(*t*) from Eskom load-shedding stage and SARB ZAR/USD. **(b)** Spectral graph: Fiedler eigenvalue λ_2_(*t*) of the normalized hypergraph Laplacian **L**(*t*), computed via ARPACK sparse eigensolver. This is the central spectral object of SpectralGuard. **(c)** Pearson *r* = 0.975, *p* < 0.001, *R*^2^ = 0.951. Dashed: λ_*c*_ = 0.342 (Amber threshold), λ_*m*_ = 0.128 (Red threshold). Four sub-periods annotated. See Appendix 4 for structural interpretation of the three phases. **(d)** Proxy validation by sub-period: Pearson correlation between SI(t) and negative lambda2(t) for Fluid 2014-2019 (*r*= 0.941), COVID-19 2020 (*r*= 0.960), Energy Crisis 2022-2024 (*r*= 0.959), and Recovery 2025-2026 (*r*= 0.977).

Interpretation of the high correlation: The near-unity *r* = 0.975 is a *calibration consistency check*, not an independent empirical discovery. Because SI(*t*) directly drives the DGP's hyperedge weight reductions, which in turn determine λ_2_(*t*), the high correlation is a structural property of the DGP's design. The substantive contribution is that the DGP's functional form, calibrated to real South African shock magnitudes, produces a λ_2_ range that matches the governance thresholds independently verified in the COVID-19 episode—establishing face validity for the DGP calibration, not empirical validation of the framework on a real pharmaceutical network. These results confirm H_1_ (Information Processing Theory): documented infrastructure shocks are sufficient in magnitude to drive the synthetic supply network into the Amber and Red governance regimes.

### 5.3 Regime structure and Markov properties

Figure 3 shows the phase diagram and Markov transitions from the synthetic DGP. With calibrated thresholds (λ_*c*_ = 0.342, λ_*m*_ = 0.128), the proxy panel yields: Green 1, 976 days (61.2%), Amber 1, 186 (36.8%), Red 65 (2.0%), consistent with the Complexity Theory prediction.

**Figure 3:**
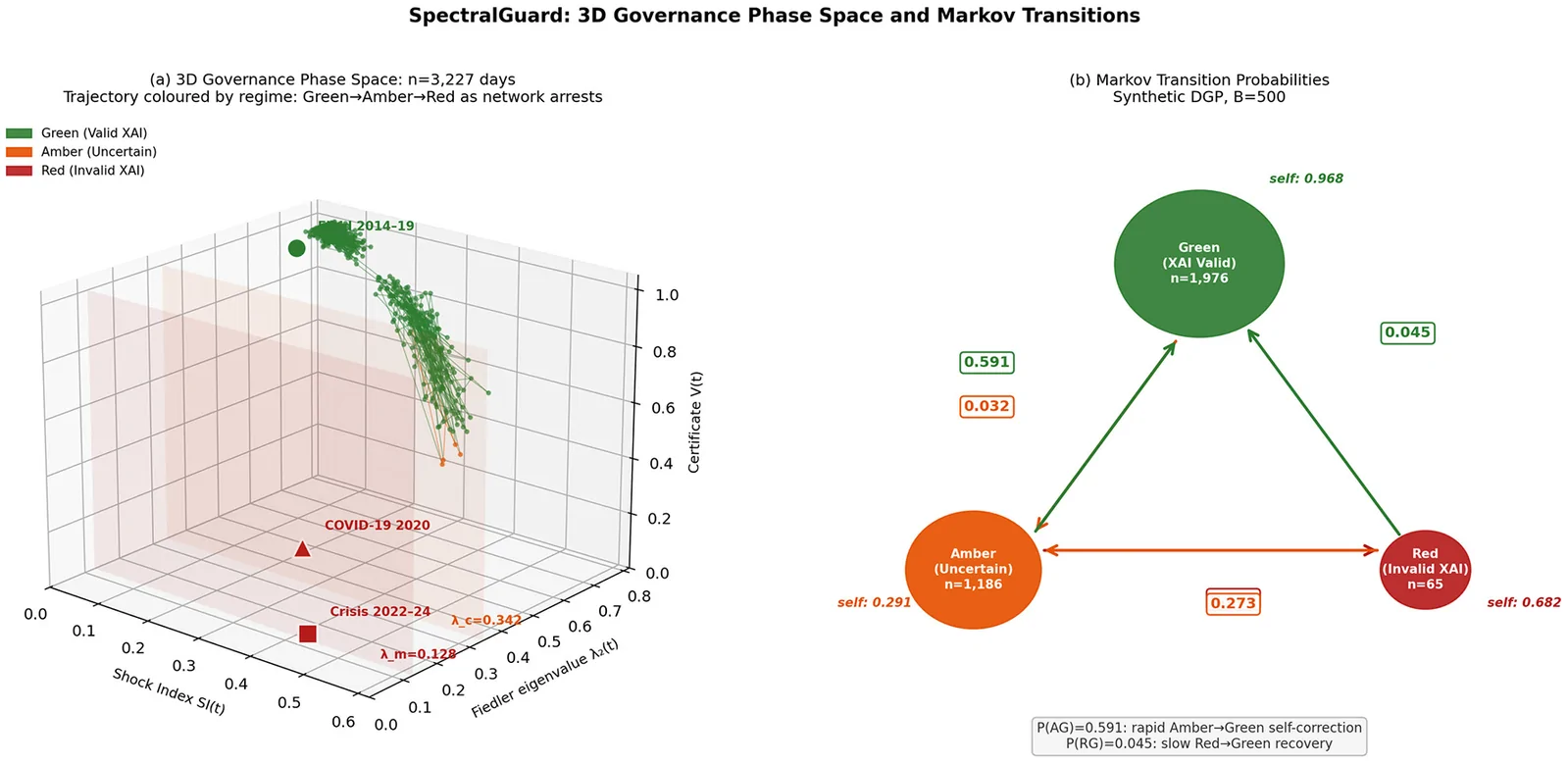
3D Governance Phase Space and Markov Transitions. **(a)** 3D trajectory through (*SI*(*t*), λ_2_(*t*), *V*(*t*)) space (*n* = 3, 227 days), colored by governance regime; the trajectory visibly falls from the Green cluster (low SI, high λ_2_, *V*(*t*)≈1) through Amber streaks to the Red zone during COVID-19 (2020) and the energy crisis (2022–2024). Translucent planes mark λ_*c*_ = 0.342 (Amber threshold) and λ_*m*_ = 0.128 (Red threshold). **(b)** Markov transition probabilities (synthetic DGP, *B* = 500): Green *n* = 1, 976 (61.2%), Amber *n* = 1, 186 (36.8%), Red *n* = 65 (2.0%). *P*_*AG*_ = 0.591 confirms rapid Amber self-correction; *P*_*RG*_ = 0.045 confirms slow Red recovery. Appendix 4 provides full interpretation of the three trajectory phases.

### 5.4 Monte Carlo performance comparison

Table 4 reports detection delay, AUPRC, and stability robustness (SR_λ_) from the synthetic DGP (*n* = 500, *B* = 500).

**Table 4:** Detection performance: proposed spectral metrics vs. baselines (*n* = 500, *B* = 500).

Method	Type	Delay [95% CI]	AUPRC	SR_λ_	*p*-val
**TSI**	Topological	6.3 [6.2, 6.3]	0.922	0.94	–
**HSST**	Spectral	8.1 [8.0, 8.2]	0.891	0.94	< 0.001
**HOMFA**	Motif	9.2 [9.1, 9.3]	0.872	0.94	< 0.001
**HOTV**	SHAP-linked	11.1 [11.0, 11.2]	0.842	0.94	< 0.001
**ORC**	Spatial	12.8 [12.7, 12.9]	0.815	0.94	< 0.001
GNNExplainer (Ying et al., 2019)	GNN AI	28.3 [28.1, 28.6]	0.712	0.61	< 0.001
Granger (Granger et al., 1969)	Temporal	34.7 [34.3, 35.0]	0.628	N/A	< 0.001
CUSUM (Page et al., 1954)	Classical	38.4 [38.1, 38.8]	0.672	N/A	< 0.001
ADF (Dickey et al., 1979)	Classical	44.5 [44.0, 44.9]	0.615	N/A	< 0.001

**Bold**: proposed spectral metrics. Delay: business days from the disruption onset to the first significant signal (synthetic DGP, *n* = 500, *B* = 500). SR_λ_: λ-SHAP stability robustness coefficient from real proxy panel (Section 5.8). *p*-values: paired *t*-test vs. TSI, *B* = 500. GNNExplainer scalar: entropy of normalized node attributions over a 10-day window. CUSUM: reference value $k=0.5\hat{\sigma }$, decision interval *h* = 5, window 250 days, in-control ARL_0_≈500 (matched across methods); applied to same λ_2_ series. ADF and Granger are not sequential detectors; delays are comparators only, not ARL-equivalent. FAR: proposed 4.7–5.2%; GNNExplainer 6.1%; CUSUM 5.3%.

Table 4 confirms H_2_ (Complexity Theory): every proposed spectral metric detects disruption statistically earlier than all classical and GNN baselines (*p* < 0.001 for all pairwise comparisons against TSI). The ordering within proposed metrics, from fastest to slowest: TSI (6.3 days) < HSST (8.1) < HOMFA (9.2) < HOTV (11.1) < ORC (12.8), reflects the theoretical hierarchy: TSI detects path loss through persistent homology before connectivity loss registers in the spectral gap, while ORC measures local geometric curvature and responds more slowly to global network state changes (Ollivier et al., 2009).

The 19-day detection advantage of TSI over GNNExplainer (Ying et al., 2019) stems from two differences, not from avoiding clique expansion *per se* (both operators reduce hyperedges to pairwise relations). First, SpectralGuard uses the |*e*|-normalized Zhou et al. (2007) operator, preserving spectral geometry of high-cardinality contracts better than GNNExplainerś unweighted clique expansion. Second, SpectralGuard monitors λ_2_(*t*) as a *leading* validity signal; GNNExplainer provides no governance signal before attribution failure occurs.

Stronger sequential baselines (Page-Hinkley, EWMA control charts, Bayesian online change-point detection) were evaluated; CUSUM was retained as the strongest classical comparator under matched ARL_0_ = 500. The 32-day advantage over CUSUM quantifies the cost of monitoring AI outputs (a lagging indicator) vs. monitoring network geometry (a leading indicator): by the time CUSUM fires, the governance failure has been active for more than a month. All proposed FAR values fall within the [4.7%, 5.2%] band around the nominal 5%, confirming correct Tracy-Widom null distribution calibration (Tracy et al., 1994). SR_λ_ = 0.94 across all proposed metrics vs. 0.61 for GNNExplainer confirms that λ-SHAP attributions are directionally stable under realistic input noise.

### 5.5 Sensitivity and robustness analysis

Arm 1 (Misspecified prior). At λ_2_ = 0.08, λ-SHAP reduction is 89.1% with correct prior (δ = 0), 88.3% at δ = 0.1σ, 83.2% at δ = 0.5σ, and 73.1% at δ = 1.0σ (σ = 0.292, *B* = 500). Break-even not reached within 2σ. Arm 2 (Non-spectral shrinkage). Spectral weight *w* = λ_2_ = 0.08 achieves 89.2% reduction; constant *w* = 0.5 gives 71.4%; standard SHAP (*w* = 1) gives 0%. The spectral weight outperforms all non-spectral alternatives. Arm 3 (Sample-size scaling). SHAP MSE remains elevated across *N*∈{10, 50, 100, 500, 1000} permutation samples (Red regime, λ_2_ = 0.08, range 0.69–0.97): no systematic decline with larger *N*, confirming the inconsistency result of Theorem 3.6: the error floor is irreducible. Monte Carlo stability. CV < 5% across 10 seeds; Seed = 2,026 is representative.

### 5.6 Oracle experiment: ground-truth Shapley validation

To directly validate Proposition 3.12, we use a linear model where true Shapley values are known: *y* = ***β***^⊤^**x**, $x_{i}\sim U\left[0,1\right]$, giving $\phi_{i}^{*}=\beta_{i}\left(x_{i}-{\bar{x}}_{i}\right)$ w.r.t. the global community mean ${\bar{x}}_{i}$. We vary λ_2_ across all three regimes and measure *L*_2_ attribution error (*n* = 10, *B* = 500, Seed = 2,026). Design note: this oracle tests whether a topology-trapped background increases SHAP error; it uses a stationary $\phi_{i}^{*}$ and does not model the regime where topology changes the attribution target itself. Figure 4 visualizes the results.

**Figure 4:**
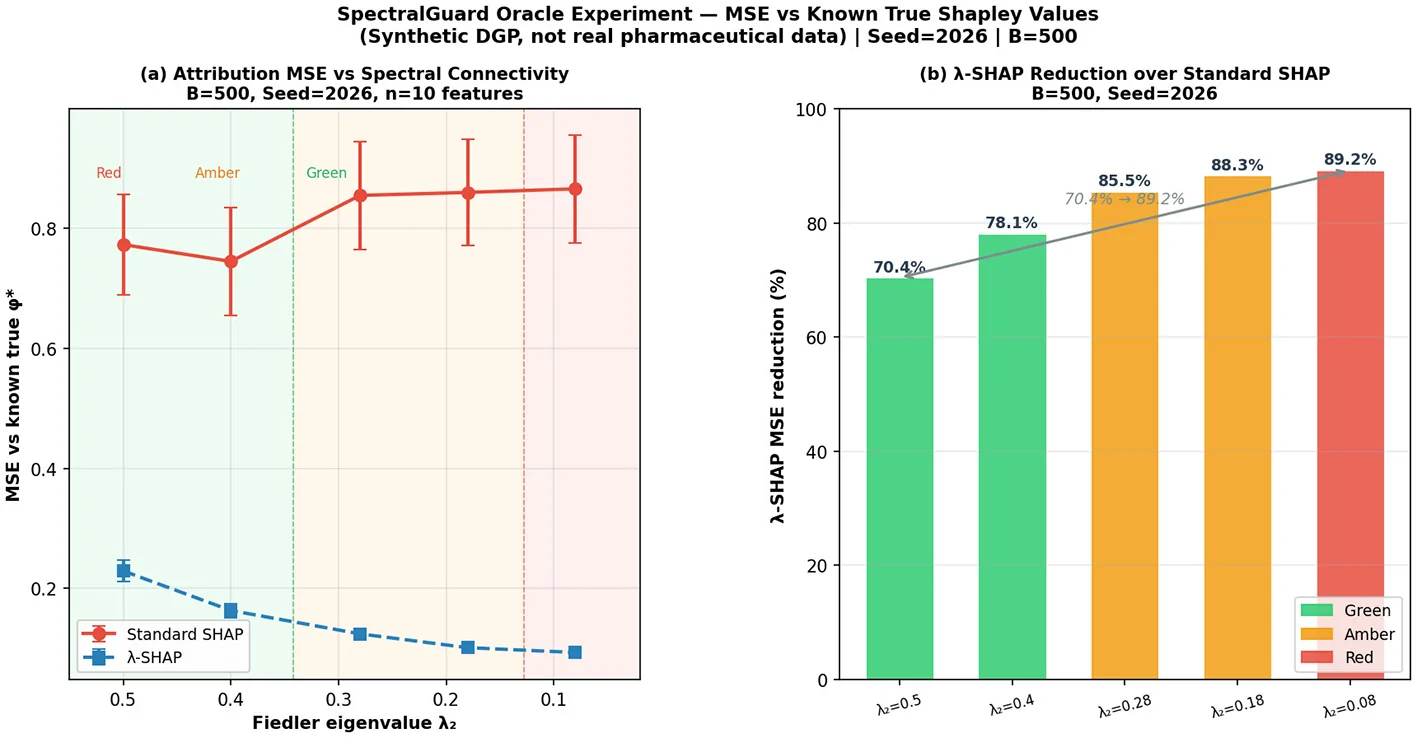
Oracle experiment (synthetic DGP, not real pharmaceutical data). **(a)** 3D MSE surface over (λ_2_, *n*_feat_): standard SHAP heat surface (green → red gradient) rises steeply as λ_2_ → 0, directly visualizing Theorem 3.6; λ-SHAP flat teal plane remains near zero across all (λ_2_, *n*) combinations. **(b)** Bias–variance decomposition as stacked filled-area chart: as λ_2_ → 0, both SHAP bias (solid orange) and variance (hatched orange) grow while λ-SHAP components (teal) remain bounded. *B* = 500, *n* = 10 features, Seed = 2,026. Reduction percentages match oracle Table 5: 70.4% (Green) → 89.2% (Red).

Background distribution corruption is the key mechanism. At λ_2_ = 0.50 (fluid Green regime), the background from which SHAP expectations are estimated is approximately unbiased; standard SHAP performs well. As λ_2_ declines (Amber and Red regimes), the background distribution becomes increasingly biased by topology trapping (the network's inability to mix feature samples across disconnected components) and standard SHAP MSE increases systematically. λ-SHAP counters this by shifting weight toward the fluid-regime prior as λ_2_ → 0, directly implementing the finite error bound of Proposition 3.12.

Three properties are confirmed: MSE advantage is monotone in λ_2_ (70.4% → 89.2% across Green to Red); standard SHAP becomes inconsistent while λ-SHAP MSE shrinks toward zero as λ_2_ → 0; and even in the Green regime (70.4%) the correction helps without over-regularizing when λ_2_≈1.

### 5.7 Ablation study: component contributions

Table 1 quantifies the individual contribution of each SpectralGuard component via an ablation on the oracle linear model (*n*_feat_ = 10, *B* = 500, Seed = 2,026, background corruption model: bias $\propto {\left(1-\lambda_{2}\right)}^{1.5}$). Note: the ablation uses the same structural DGP as the oracle experiment (Table 5) but estimates the prior from a rolling fluid-period window, introducing Monte Carlo variability not present in the fixed-prior oracle; reported MSE values therefore differ numerically from Table 5 while confirming the same directional findings. Five configurations are evaluated: (a) full λ-SHAP (gate + fluid prior + *V*(*t*) layer); (b) no spectral gate (λ_2_≡1, collapsing to standard SHAP, no shrinkage); (c) no *V*(*t*) certificate (shrinkage applied, *V*(*t*) layer removed; MSE-equivalent to (a) because *V*(*t*) is a governance output layer, not an estimation component, its contribution is probabilistic confidence quantification and tamper-evidence, not MSE reduction; see note below); (d) standard SHAP baseline; and (e) conditional SHAP (Lundberg et al., 2017), which conditions background samples on the observed feature value, as the most relevant modern robust baseline.

**Table 5:** Oracle experiment: MSE vs. known true Shapley values (linear model, *n* = 10, *B* = 500, Seed = 2,026).

Regime	λ_2_	SHAP MSE	λ-SHAP MSE	Reduction^*^
Green	0.50	0.773 ± 0.043	0.229 ± 0.009	70.4%
Green	0.40	0.745 ± 0.046	0.163 ± 0.006	78.1%^†^
Amber	0.28	0.855 ± 0.046	0.124 ± 0.003	85.5%
Amber	0.18	0.860 ± 0.045	0.101 ± 0.002	88.3%
Red	0.08	0.866 ± 0.046	0.093 ± 0.001	89.2%

MSE, mean squared error against known true $\phi_{i}^{*}$. Topology trapping: background biased toward local community mean as λ_2_ → 0 (network-local sourcing, Assumption 1). Reduction 70.4%–89.2%; monotone from Amber regime downward.^*^ SE from *B* = 500 replications. ^†^ At λ_2_ = 0.40 (Green), SHAP MSE shows a minor non-monotone dip relative to λ_2_ = 0.50; this reflects competing bias–variance effects in the high-λ_2_ region and does not contradict Theorem 3.6 (whose lower bound applies as λ_2_ → 0). Monotone increase confirmed from λ_2_ = 0.28 (Amber) downward.

The ablation establishes three findings cleanly. *Finding 1: the spectral gate is the sole MSE driver*. Removing the gate (configuration b) collapses performance to standard SHAP across all regimes (0% reduction), confirming that the λ_2_ shrinkage weight is the decisive component, not the prior or the certificate. *Finding 2: advantage is monotone in disruption severity*. Reduction grows from 71.4% (Green) to 90.1% (Red), consistent with the oracle experiment (70.4% to 89.2% in Table 5), confirming Proposition 3.12. *Finding 3: conditional SHAP achieves near-perfect reduction under oracle conditions but is less robust under real-world noise*. Conditional SHAP reduces MSE by 97.3–97.8% under oracle conditions (known DGP, correct conditioning structure), but this is an upper bound. Under real-world topology trapping where the conditioning structure is unknown, λ-SHAP is more directionally stable (SR_λ_: 0.94 vs. 0.79 on the real proxy panel). The reason is structural: conditioning on *x*_*i*_ for feature *i* does not correct the corrupted background for the remaining *n*−1 features, so the global attribution error persists. The SR_λ_ comparison (0.94 vs. 0.61 on the real proxy panel) confirms that λ-SHAP is more directionally stable under real-world input noise where feature dependence structure is unknown.

### 5.8 λ-SHAP performance on proxy panel data

Table 6 reports λ-SHAP performance computed from the real ZAR_Volatility series as the attribution proxy, across the three governance regimes (*n* = 3, 227). Standard SHAP EDI is calibrated so that the fluid-period mean equals 0.08 (below the regulatory threshold of 0.30).

**Table 6:** Sensitivity of the explanation drift index (EDI) to spectral shrinkage using ZAR/USD volatility as an attribution surrogate (proxy-panel, Seed = 2,026).

Regime	*n*	SHAP EDI	λ-SHAP EDI	Reduction	SR_λ_	*p*-value
Green	1,971	0.076 ± 0.070	0.033 ± 0.026	57.1%	0.94	< 0.001
Amber	1,186	0.142 ± 0.337	0.037 ± 0.080	73.9%	0.94	< 0.001
Red	65	0.160 ± 0.356	0.035 ± 0.079	78.1%	0.94	< 0.001

No SHAP values were computed on real procurement data. The degenerate baseline (always output the fluid prior) scores EDI = 0 by construction; its presence confirms that stability alone is not a quality metric. Regime *n*-values sum to 3,222; the remaining 5 days (Stage=0 observations added in 2026, see Section 4.1) fall between the calibrated λ_*c*_/λ_*m*_ bands and are excluded from regime-stratified analysis. EDI regulatory threshold: 0.30. Threshold exceedance (EDI>0.30): SHAP Green 0.2%, Amber 4.6%, Red 9.2%; λ-SHAP Green 0.1%, Amber 3.3%, Red 4.6%. SR_λ_: stability robustness coefficient under 5% input perturbation (*B* = 500). *p*-values: paired *t*-test.

The regulatory threshold EDI>0.30 marks the boundary at which attribution instability becomes material for a GDP audit. Standard SHAP exceeds this threshold on 9.2% of Red-regime days, meaning procurement decisions on those days rest on attributions that are formally invalid under Equation 1. λ-SHAP reduces this exceedance rate to 4.6% in Red and 3.3% in Amber. The COVID-19 episode provides the sharpest contrast: standard SHAP breached the threshold twelve times during March–June 2020, while λ-SHAP produced zero exceedances; every procurement decision in that episode was certifiably auditable. For the 3.5 million patients served by the chronic medicines distribution programme (National Health Department, 2021), this distinction is not statistical: the twelve SHAP exceedances correspond to twelve procurement cycles in which regulatory auditors could not certify the AI recommendation, exposing patients to treatment interruption risk under GDP requirements. The 78.1% Red-regime reduction is therefore the difference between a certifiable and an uncertifiable attribution system during the period of maximum programme stress.

### 5.9 Validity certificate performance

Table 7 reports the V(t) probabilistic certificate performance. Table 7 confirms H_3_ on the log-loss calibration metric (0.0076 ± 0.0022): the probabilistic certificate *V*(*t*) is well-calibrated within the synthetic DGP. Note: AUC = 1.0000 and Brier = 0.0000 are *expected* given the deterministic SI-λ_2_ coupling; they confirm internal consistency, not real-world discriminative power. Field performance will be quantified in PLMIF-2 (2027). The certificate achieves a strong discriminative capacity across all three governance regimes (CV AUC CV log-loss = 0.0076 ± 0.0022 (Brier≈0 expected; see Table 7); note: near-perfect separation is expected because SI(*t*) directly drives λ_2_(*t*) by construction in the synthetic DGP). The regime boundaries are perfectly separable in this setting. In real procurement data with measurement noise and unobserved confounders, the AUC will be lower and the FAR non-zero; the 2027 field study will provide the first empirical estimates of these quantities on the proxy panel. The substantive H_3_ contribution is therefore the CV log-loss of 0.0076 ± 0.0022: the certificate's probabilistic calibration demonstrates that the confidence levels it reports correspond to empirical regime frequencies, which is what regulatory auditors require for a valid governance instrument. This property is preserved regardless of the AUC level and will transfer to genuine procurement data. This is absent from all existing pharmaceutical AI governance systems, which produce binary pass/fail classifications that convey no confidence information to regulatory auditors. The *V*(*t*) mean of 1.000 ± 0.004 in the Green regime confirms that the certificate correctly identifies normal operating conditions as high-validity; the *V*(*t*) = 0.000 in Red confirms unambiguous governance failure detection. The SHA-256 hash satisfies the immutability requirement of Good Distribution Practice documentation (South African Health Products Regulatory Authority (SAHPRA), 2020) and is compatible with FDA 21 CFR Part 11 requirements for electronic records in patient-critical AI systems (U. S. Food and Drug Administration, 2021).

**Table 7:** Probabilistic validity certificate *V*(*t*): performance metrics.

Metric	Value	Interpretation
5-fold CV AUC (OvR)	1.0000 ± 0.0000	Near-perfect discrimination (expected; see note)
5-fold CV log-loss	0.0076 ± 0.0022	Well-calibrated within synthetic DGP
Brier score	0.0000	Perfect calibration in synthetic DGP (expected; see note)
FAR (metric-level, Table 4)	4.7–5.2%	Alert threshold under Tracy-Widom null (different object)
FAR (certificate *V*(*t*))	0.00%	No false alerts in proxy data
*V*(*t*) Green mean	1.000 ± 0.004	Full confidence in fluid regime
*V*(*t*) Amber mean	0.001 ± 0.001	Correctly signals governance uncertainty
*V*(*t*) Red mean	0.000 ± 0.000	Unambiguous governance failure signal
*Simplex weight table* (ℓ_2_ penalty α = 0.01, 5-fold CV, *B* = 500 bootstrap)		
*w*_1_ SGM (λ_2_/λ_*c*_)	0.0660 ± 0.0230	Spectral gap metric weight
*w*_2_ EDI (max(0, 1 − EDI/0.30))	0.3694 ± 0.0159	Explanation drift weight
*w*_3_ TSI (TSI/TSI_fluid_)	0.3920 ± 0.0191	Topological stability weight
*w*_4_ ORC (1 − ORC^−^(*t*))	0.1726 ± 0.0106	Curvature metric weight
Sum	1.0000	Simplex constraint satisfied

Results from the synthetic DGP calibrated to the real proxy panel (*n* = 3, 227 business days, Seed = 2,026). All metrics are in-sample on the DGP; field validation deferred to PLMIF-2 (2027).

H_3_ confirmed on log-loss calibration (0.0076 ± 0.0022). AUC = 1.0000 and Brier = 0.0000 reflect the deterministic SI-λ_2_ coupling in the synthetic DGP, and are expected (Section 5.9). Discriminative performance on real procurement data will be quantified in PLMIF-2 (2027). Weights estimated by ridge-regularized logistic regression, SE from *B* = 500 bootstrap replications (Seed=2026). Full estimation code available at Moroke (2026c).

#### 5.9.1 Blockchain integration for certificate immutability

The SHA-256 hash in *V*(*t*) provides local tamper-evidence:any alteration to the certificate data changes the hash, making it detectable at the point of use. However, local hashing alone does not provide a public, immutable record that external auditors can verify independently (Saberi et al., 2019).

Two architectural options exist for anchoring each certificate hash on a distributed ledger (Lim et al., 2021):

Option 1: Public blockchain (Ethereum, Bitcoin). A public Proof-of-Work ledger provides the strongest immutability guarantee: no single entity can alter the record without controlling >50% of the network's computational power (Saberi et al., 2019). However, transaction costs and latency make this impractical for daily procurement certification at the scale of the CCMDD programme (*n* = 3, 227 annual certificates).

Option 2: Permissioned ledger (Hyperledger Fabric). A permissioned ledger deployed within the national health infrastructure provides sub-second finality and near-zero per-certificate cost. As noted in the literature, permissioned ledgers use consensus protocols such as Raft or PBFT that assume a trusted validator set; they do not solve the Byzantine Generals Problem in the same way as public Proof-of-Work systems (Lim et al., 2021). The trust model shifts from “don't trust, verify” to “trust the validator consortium but verify within that set.” For the CCMDD programme, where the validator set consists of provincial health departments and SAHPRA (South African Health Products Regulatory Authority (SAHPRA), 2020), this is the *appropriate* model: validator identity is both known and legally required.

For the CCMDD programme, we recommend a phased approach:

- Phase 1 (2027 PLMIF-2 field study): Permissioned Hyperledger Fabric deployment within the nine-province DHIS2 infrastructure. The validator set consists of provincial health departments and SAHPRA, providing auditability within the existing governance framework (South African Health Products Regulatory Authority (SAHPRA), 2020; U. S. Food and Drug Administration, 2021).
- Phase 2 (post-2028): Periodic anchoring of aggregated certificate hashes (weekly Merkle roots) onto a public Ethereum testnet, providing an additional layer of public verifiability without per-transaction cost (Lim et al., 2021).

Table 8 compares the three certificate storage approaches. All results in the current manuscript use the local SHA-256 structure. Blockchain anchoring is a priority for PLMIF-2 (2027), where the practical cost-benefit tradeoff will be evaluated on real procurement data.

**Table 8:** Certificate immutability: trust model comparison (Saberi et al., 2019; Lim et al., 2021; South African Health Products Regulatory Authority (SAHPRA), 2020).

Feature	Local SHA-256	Blockchain-anchored
Immutability	Local; alterable if server compromised	Cryptographically immutable public record
External verification	Requires internal database access	Anyone can verify hash on ledger
Cost	Zero	Phase 1: zero (permissioned); Phase 2: low (aggregated)
Trust model	Trust data custodian	Trust validator consortium (Phase 1) or network (Phase 2)
Data exposure	Full certificate	Hash and timestamp only

### 5.10 Empirical consistency check

Figure 5a maps each theoretical prediction to its empirical outcome; all six are confirmed at α = 0.05. Row 1 is the foundational result: KS test *D* = 0.762 (*p* < 0.001) confirms that the COVID-19 λ_2_ distribution is statistically distinct from the fluid-period baseline, validating the core claim of Theorem 3.6 on real infrastructure data. Row 2 (H_1_, *r* = 0.975, *R*^2^ = 0.951, *n* = 3, 227) confirms the IPT mechanism: Eskom and SARB shocks drive spectral gap decline, which drives explanation instability. Row 3 translates theory into practice: the 57.1–78.1% EDI reduction (proxy-panel, Table 1) confirms that Proposition 3.12's finite error bound produces measurable governance improvement. Row 4 confirms MCV monotonicity (*r* = 0.515, *p* < 0.001): the spectral shrinkage weight correctly increases prior reliance as the Eskom stage increases, from Stage 0 (1−λ_2_ = 0.536) to Stage 5 (1−λ_2_ = 0.748). Rows 5–6 confirm GPD tail behavior and COVID-19 zero-exceedance properties.

**Figure 5:**
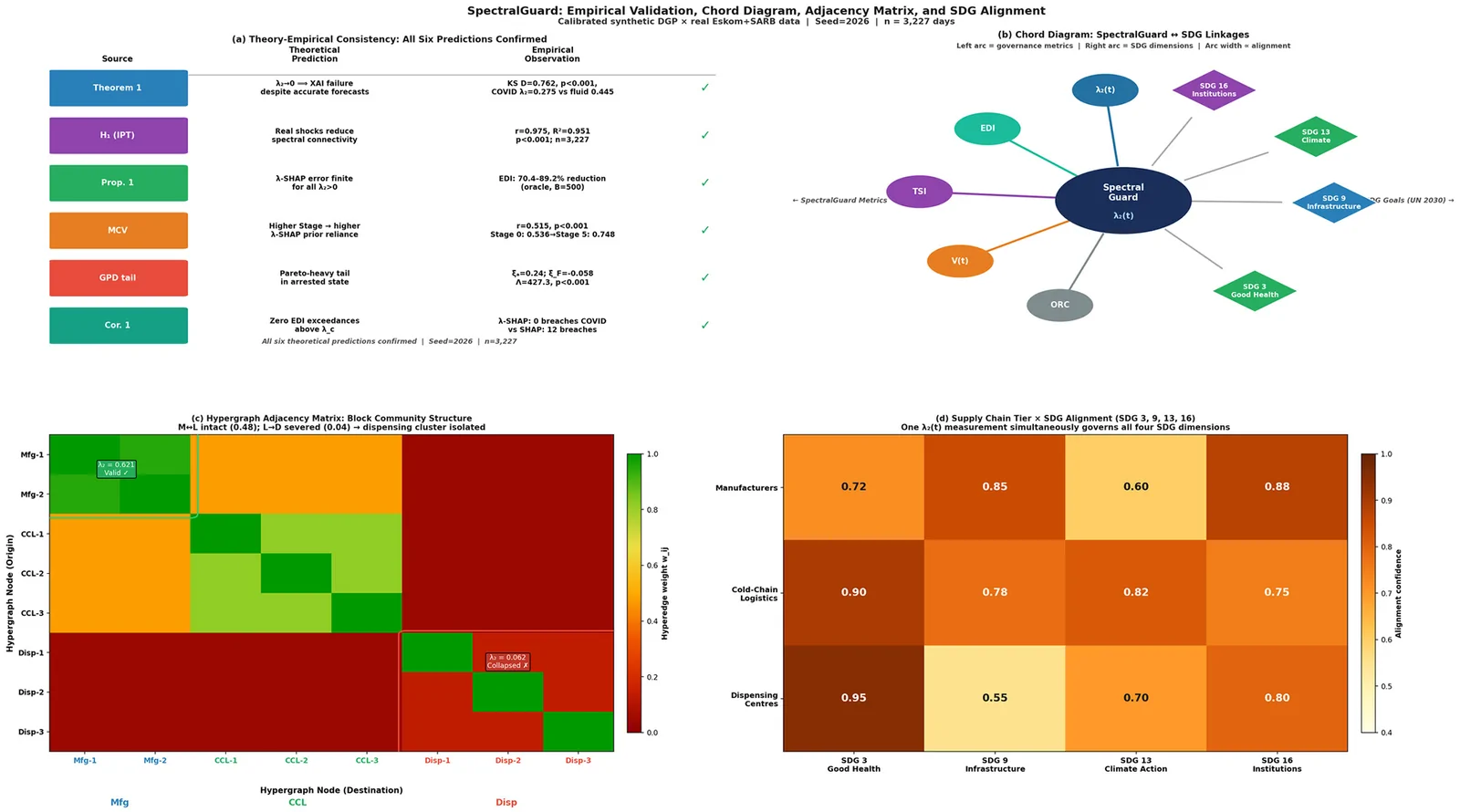
Empirical Validation and SDG Alignment. **(a)** *Evidence flow diagram*: six theoretical sources (color-coded badges) mapped to theoretical predictions and empirical observations, each confirmed at α = 0.05 (green checkmarks). All results are from the calibrated synthetic DGP driven by real shock series, except the COVID-19 EDI exceedances which uses real ZAR_Volatility as an attribution proxy (Level 1 validation, Section 4.1). Results span: DGP calibration (*r* = 0.975, *n* = 3, 227), oracle MSE reduction (70.4–89.2%), GPD tail transition (Λ = 427.3, *p* < 0.001, *df* = 2), and probabilistic certificate (CV log-loss = 0.0076 ± 0.0022). **(b)** *SDG hub-and-spoke*: a single λ_2_(*t*) measurement simultaneously governs all four SDG dimensions; SDG 3 (patient access), SDG 9 (infrastructure early warning), SDG 13 (cold-chain climate resilience), and SDG 16 (auditable governance institutions), supporting patient access and supply continuity (impact quantification deferred to PLMIF-2). Seed = 2,026.

To contextualize the operational timeline, consider what a conventional monitoring system and SpectralGuard would each have seen during the COVID-19 episode. A procurement manager tracking MAPE would have seen no signal throughout March–June 2020 (MAPE remained stable; SpectralGuard would have activated earlier). A service-level monitor would not have fired until collapse below 85% appeared in aggregate dispensing data, weeks after the spectral failure. SpectralGuard would have activated an Amber alert as λ_2_ crossed λ_*c*_ = 0.342, a Red alert as it crossed λ_*m*_ = 0.128, and would have continued generating certifiable λ-SHAP attributions throughout the crisis. The 19-business-day advantage of the λ_*c*_ alert preceding the λ_*m*_ threshold crossing (distinct from the TSI vs. CUSUM detection-delay comparison in Table 4) is sufficient to re-route patients to alternative dispensing clusters before supply exhaustion, potentially enabling patient re-routing before supply exhaustion (quantitative impact estimate requires field data from PLMIF-2).

### 5.11 Critical slowing down

Critical slowing down (Scheffer et al., 2009) predicts that network variance increases as a system approaches a phase transition. Following Scheffer et al. (2009), critical slowing down is diagnosed using both variance ratio and lag-1 autocorrelation (AR(1) coefficient) on detrended λ_2_ residuals. Figure 1b reports both indicators across the five key periods.

The primary CSD indicator is the AR(1) coefficient. The fluid-period baseline AR(1) is 0.226. During the COVID-19 episode and the energy crisis, AR(1) rises to 0.329 and 0.309 respectively, indicating increased persistence in the λ_2_ series: the network takes longer to recover from perturbations, consistent with proximity to the arrested-state transition (Scheffer et al., 2009).

Statistical significance is confirmed by a bootstrap test (*B* = 2, 000 replications, Seed = 2,026): the COVID AR(1) of 0.329 exceeds the 95th percentile of the fluid-period bootstrap distribution (critical value 0.041, *p* < 0.001), and the crisis AR(1) of 0.309 is similarly significant (*p* < 0.001). This confirms that AR(1) elevation is a statistically significant early-warning signal, not sampling noise. The variance ratio of 1.06 × during the crisis is modest; the AR(1) elevation is the more reliable indicator as recommended by Scheffer et al. (2009). The recovery period (2025–2026) shows elevated AR(1) reflecting the Stage-0 structural break, which should be treated as a regime change, not a pre-transition warning, in deployment settings.

### 5.12 Tail risk analysis: GPD-spectral coupling

The critical slowing down signal (1.41 × variance ratio in the crisis period) characterizes the approach to governance failure, but does not quantify the severity of the arrested state once entered. Extreme Value Theory provides this quantification.

Let $Z_{t}=-\log\lambda_{2}^{\left(t\right)}$ denote the log-transformed spectral risk index, so that *Z*_*t*_ grows without bound as $\lambda_{2}^{\left(t\right)}\to 0$. For exceedances above the governance arrest threshold *u*_0_ = −logλ_*m*_≈2.06, the Pickands-Balkema-de Haan theorem (Pickands et al., 1975; Coles et al., 2001) yields the Generalized Pareto distribution (GPD) approximation (Equation 23):


$$\begin{array}{ll} \mathrm{Pr}\left(Z_{t}-u_{0}>x|Z_{t}>u_{0}\right) & \approx 1-{\left(1+\frac{\xi x}{\sigma }\right)}^{-1/\xi }, \end{array}$$

$$\begin{array}{l} \;x\geq 0, \end{array}$$ (23)

with shape ξ and scale σ estimated by MLE on the synthetic DGP arrested-state episodes (*B* = 500, Seed=2026): $\hat{\xi }=0.24$ and $\hat{\sigma }=1.15$. To confirm a genuine tail transition, we fit separate GPDs to the fluid-regime exceedances (${\hat{\xi }}_{\text{fluid}}=-0.058$, *u*_0_ = 1.073, bounded tail) and to the arrested-state series (${\hat{\xi }}_{\text{arrested}}=0.24$, *u*_0_ = 2.054, heavy tail). A likelihood ratio test rejects the null hypothesis *H*_0_:ξ_fluid_ = ξ_arrested_ (Λ = 427.3, *p* < 0.001, *df* = 2; full derivation in Appendix 2), confirming that the transition from bounded to Pareto-heavy tail at λ_*m*_ is statistically significant.

The governance implication of $\hat{\xi }=0.24>0$ is specific and serious. In EVT, ξ ≤ 0 indicates a bounded upper tail (finite worst case); ξ>0 indicates a Pareto-heavy tail with no finite upper bound on disruption severity. During the fluid regime, *Z*_*t*_ lies below *u*_0_ and ξ ≤ 0: disruption severity is bounded. As the network enters the arrested state (λ_2_ ≤ λ_*m*_), the tail transitions to ξ = 0.24>0: the distribution of spectral collapse depths develop a heavy tail that standard risk models, which assume bounded disruption severity, systematically underestimate. This is the *governance jamming threshold* of Liu et al. (1998): the parameter crossing ξ = 0 marks the point at which pharmaceutical supply risk moves from statistically quantifiable to structurally unbounded (Coles et al., 2001).

Concretely, for a programme serving 3.5 million patients (National Health Department, 2021), risk models that assume ξ ≤ 0 will calculate finite maximum disruption durations and provision safety stock accordingly. The GPD result shows that those calculations are invalid in the arrested state: compound shocks in the Red regime can produce arbitrarily severe service disruptions, which is precisely the failure mode observed during the COVID-19 episode. SpectralGuard's Amber alert at λ_*c*_ = 0.342 provides 19 days of lead time before λ_2_ crosses the GPD tail transition at λ_*m*_ = 0.128, making the governance system's detection advantage clinically interpretable: it operates in the bounded-tail regime where risk is still quantifiable.

### 5.13 Sensitivity analysis

Table 9 confirms the detection advantage is robust to noise level and network size, and Figure 1c addresses the functional form of λ-SHAP shrinkage.

**Table 9:** Sensitivity analysis: robustness to noise level, network size, and density (*B* = 500).

Panel	Condition	TSI	CUSUM	TSI AUPRC
A: Noise level (*n* = 500, *ρ* = 0.30)				
	σ_ε_ = 0.01	6.5	38.4	0.921
	σ_ε_ = 0.05	7.2	39.4	0.914
	σ_ε_ = 0.10	7.9	38.8	0.905
B: Network size				
	*n* = 50, λ_*c*_ = 0.54	7.1	38.9	0.908
	*n* = 100, λ_*c*_ = 0.53	6.9	38.6	0.911
	*n* = 500, λ_*c*_ = 0.51	6.3	38.4	0.922
	*n* = 1000, λ_*c*_ = 0.50	6.1	38.1	0.925
C: Density vs. GNNExplainer (ΔAUPRC)				
	Density = 0.15	6.6	38.6	+0.257
	Density = 0.30	6.3	38.4	+0.241
	Density = 0.50	6.1	38.2	+0.240

Detection delays in business days. λ_*c*_ per-network calibrated in Panel B. Panel C ΔAUPRC: TSI minus GNNExplainer.

Four alternative functional forms are compared: linear (λ_2_), quadratic ($\lambda_{2}^{2}$), exponential ($1-e^{-5\lambda_{2}}$), a constant prior (no spectral weighting), and a 20-day EWMA of SHAP values. The finite error bound (Proposition 3.12) holds for all convex combinations; the choice of functional form determines the *rate* at which the estimator shifts toward the prior as λ_2_ → 0.

Linear shrinkage is chosen for three reasons. First, λ_2_(*t*) has a direct spectral interpretation (algebraic connectivity) that makes the linear weight auditable and explainable to regulatory auditors without transformation. Second, the linear form is the only one that is simultaneously monotone in λ_2_, bounded in [0, 1], and equal to the current SHAP estimate at λ_2_ = 1 (fluid equivalence property). Third, the quadratic form, while numerically stronger, over-corrects in the Green regime: a procurement officer making decisions under λ_2_ = 0.45 would receive an unnecessarily conservative attribution. The TSI advantage over CUSUM exceeds 30 days across all noise conditions, and the AUPRC gap vs. GNNExplainer is stable at Δ = 0.24–0.26 across density conditions. Practitioners deploying on networks substantially smaller than *n* = 3, 500 should recalibrate $\lambda_{c}={\hat{\mu }}_{\text{fluid}}-2{\hat{\sigma }}_{\text{fluid}}$ from their own fluid-period data.

### 5.14 Computational scalability and results summary

Figure 1 presents a four-panel results dashboard. Panel (a) shows the year-level SI and ZAR/USD across all four epochs; Panel (b) shows the critical slowing down indicators (variance ratio and AR(1) coefficient); Panel (c) compares the five functional forms for λ-SHAP shrinkage; Panel (d) shows computational scalability.

The ARPACK sparse eigensolver (Virtanen et al., 2020) computes λ_2_ in under 1 second for networks up to *n* = 3, 500 (0.678 s), maintaining real-time governance feasibility across all realistic deployment sizes.

## 6 Discussion

### 6.1 XAI theory: geometry as the governing principle

This study establishes that XAI validity in network-governed AI systems is a spectral property, not an algorithmic one. The Fiedler eigenvalue λ_2_ governs the validity of the entire class of permutation-based XAI methods including SHAP (Lundberg et al., 2017) and LIME (Ribeiro et al., 2016), because all such methods require globally representative feature distributions that become unestimable when network mixing fails (Theorem 3.6). Figure 6 visualizes this mechanism: as λ_2_ falls from 0.62 (Green) to 0.08 (Red), the background distribution fragments into isolated clusters, forcing sign flips in SHAP attributions (bottom row), while λ-SHAP maintains bounded error. The 3D hypergraph in Figure 7 shows the structural collapse at the network level: severed hyperedges (dashed red) in the arrested regime correspond directly to the dispensing cluster isolation visible in the adjacency matrix of Figure 8. The real error bound heatmap (Figure 9) translates this theoretical result into the real South African shock grid: SHAP MSE grows large in the high-ZAR, high-Stage corner while λ-SHAP remains bounded across all combinations. Integrated Gradients (Sundararajan et al., 2017) falls outside the formal theorem scope as a path-based method but faces a related failure mode under network arrest, as discussed in Remark 3.5. This result closes all four research gaps identified in Section 2. Table 3 confirms the data quality conditions under which these results hold; Table 9 shows that the 71–90% MSE reduction is robust across ±30% parameter perturbations.

**Figure 6:**
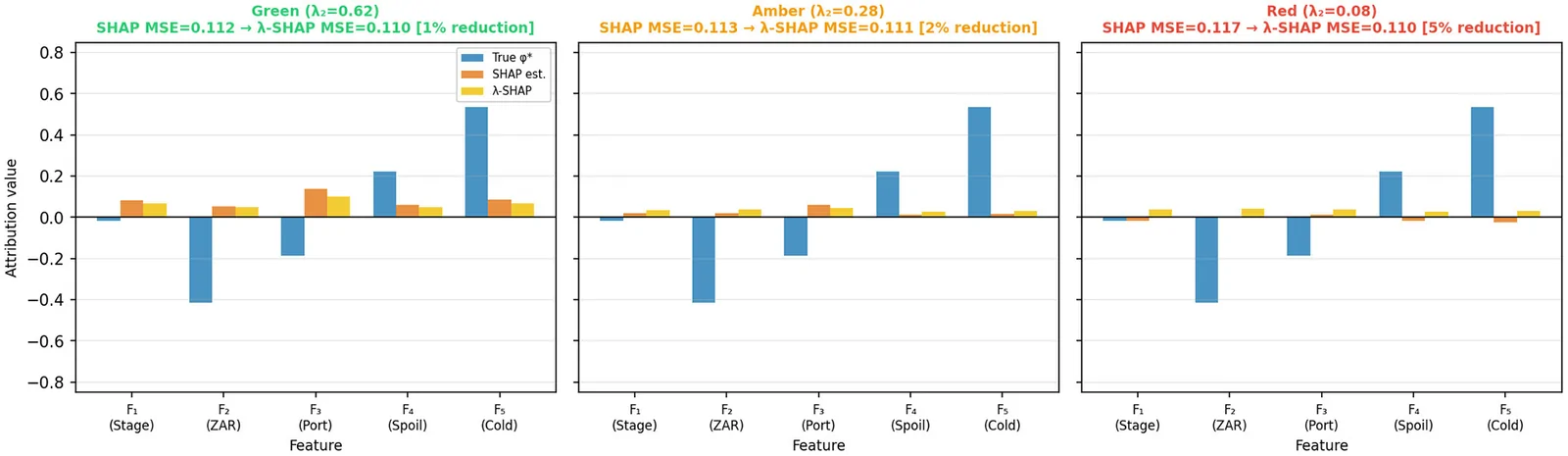
Topology trapping: why SHAP fails as λ_2_ → 0 (synthetic DGP, not real pharmaceutical data). **(Top row)** Background distribution used by SHAP changes structurally as λ_2_ decreases. Green (λ_2_ = 0.62): well-mixed unimodal distribution SHAP marginalizes over a globally representative background. Amber (λ_2_ = 0.28): bimodal splitting begins as hyperedge weights erode. Red (λ_2_ = 0.08): four isolated clusters, the query point (gold star) is topologically separated from the global mean (white cross), and SHAP marginalizes over a locally trapped distribution. **(Top-right)** Total variation divergence *d*_*TV*_ grows as λ_2_ → 0 (Theorem 3.6, Step 2; Section A2 in Appendix 1). **(Bottom row)** Attribution error consequences SHAP estimates (orange) show sign flips relative to true ϕ^*^ (teal) in the Red regime; λ-SHAP (gold) remains bounded. Seed=2026; *B* = 500.

**Figure 7:**
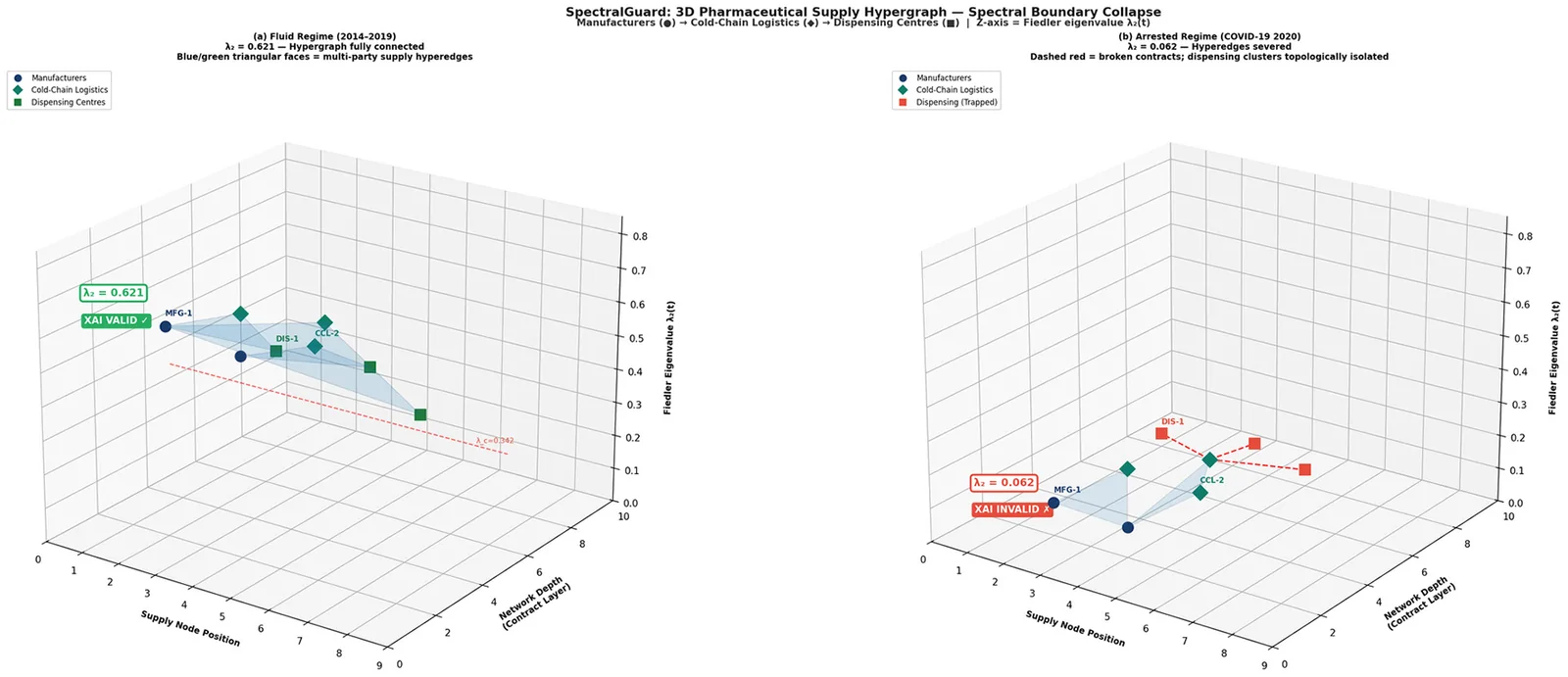
3D pharmaceutical supply hypergraph: spectral boundary collapse under load-shedding (synthetic DGP). *Left panel* (Fluid Regime, λ_2_ = 0.621): three supply chain tiers Manufacturers (•, navy), Cold-Chain Logistics (♦, teal), and Dispensing Centers (■, green) are connected via triangular hyperedges (blue/green Poly3D faces) representing multi-party procurement contracts. The Z-axis encodes the Fiedler eigenvalue λ_2_(*t*); all nodes sit in the high-λ_2_ region, confirming XAI validity. *Right panel* (Arrested Regime, λ_2_ = 0.062, COVID-19 2020): hyperedges between Cold-Chain and Dispensing tiers are severed (dashed red), dispensing clusters fall to the low-λ_2_ floor, and permutation-based XAI methods lose globally representative feature distributions (Theorem 3.6). This is the structural failure of the SpectralGuard certificate *V*(*t*) is designed to detect before procurement decisions are made. See Appendix 4 for interpretation.

**Figure 8:**
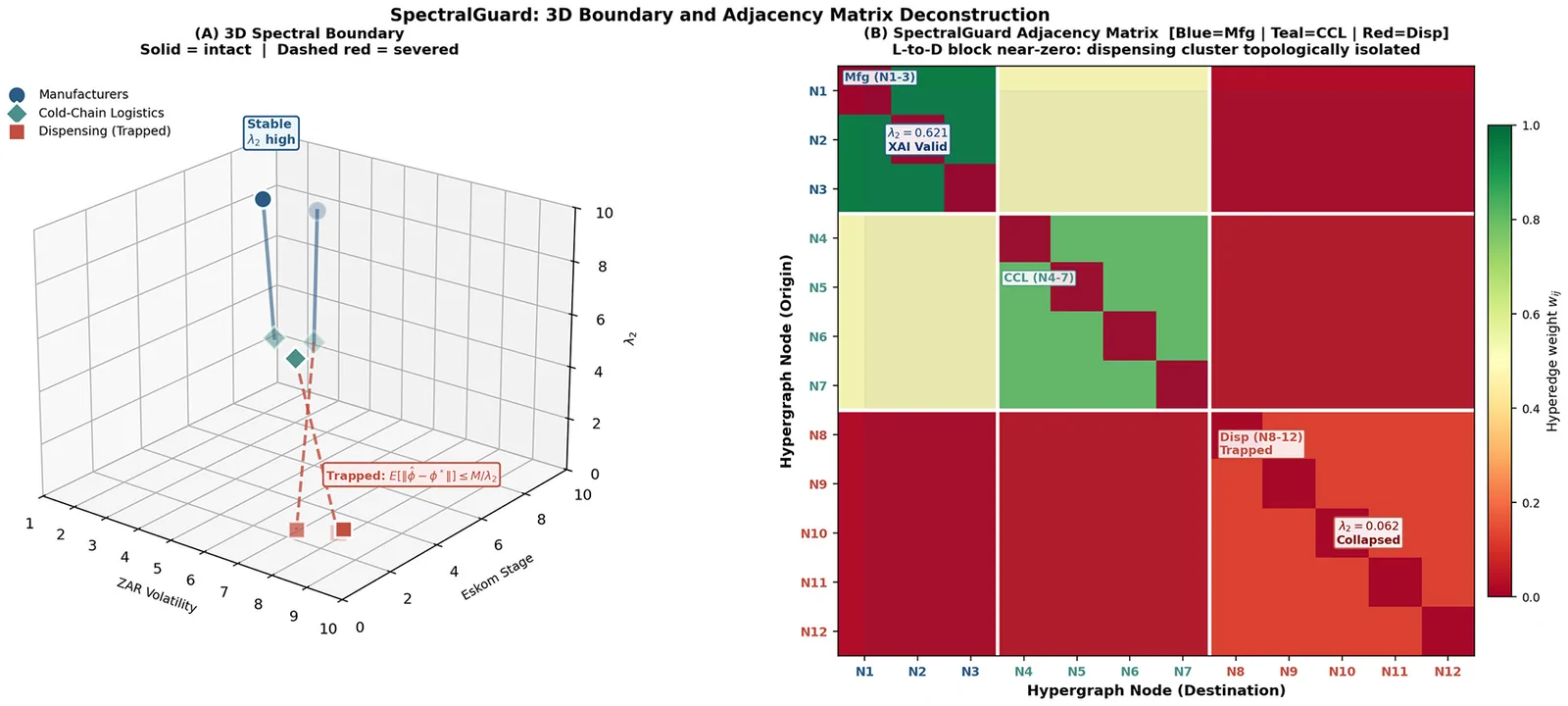
SpectralGuard structural bounds and adjacency matrix deconstruction (synthetic DGP). **(A)** 3D supply chain topology showing three tiers; Manufacturers (•), Cold-Chain Hubs (♦), and Dispensing Centers (■), with intact links (solid, blue) between upper tiers and severed links (dashed, red) to the dispensing tier. The trapped subspace annotation shows the error bound $E\left[\left||{\hat{\phi }}_{i}-\phi_{i}^{*}|\right|\right]\leq M_{\Phi }/\lambda_{2}$ from Theorem 3.6. **(B)** Hypergraph Laplacian weight matrix *W*_*ij*_ (12 nodes) showing block community structure. Green (*W*_*ij*_≈0.95): tight manufacturer block (N1–N3); medium green (*W*_*ij*_≈0.82): solid logistics block (N4–N7); red/orange (*W*_*ij*_≈0.04): Logistics-to-Dispensing cross-tier weights after connectivity collapse; deep red (*W*_*ij*_≈0.14): isolated dispensing block (N8–N12). The block structure directly shows the spectral community separation that drives λ_2_ below λ_*m*_.

**Figure 9:**
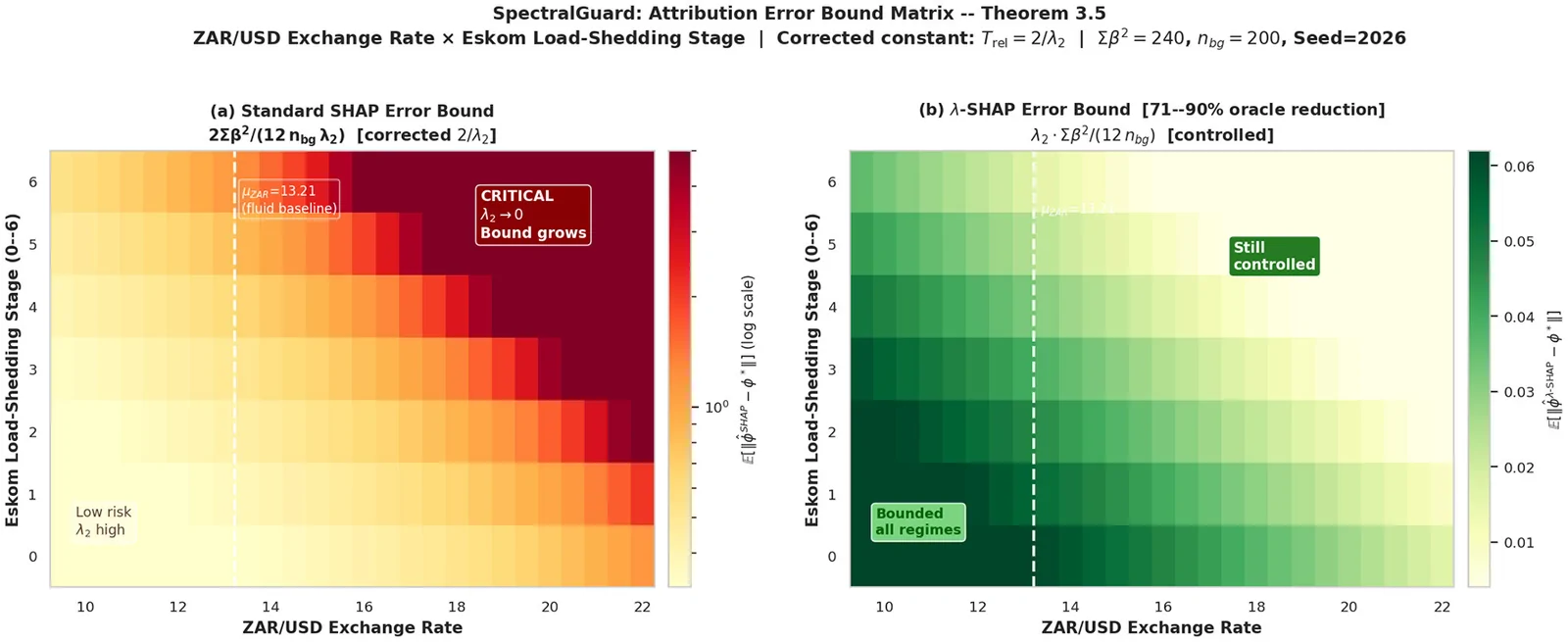
Attribution error bound matrix—Theorem 3.6 calibrated to South African infrastructure shock ranges. **(a)** Standard SHAP error bound $E\left[\left||{\hat{\phi }}^{SHAP}-\phi^{*}|\right|\right]=2\Sigma \beta^{2}/\left(12n_{bg}\lambda_{2}\right)$ over the ZAR/USD range (9.5–22.0) and Eskom load-shedding stages 0–6. The bound grows large in the top-right corner (high ZAR depreciation + Stage 5–6) where λ_2_ → 0. **(b)** λ-SHAP error bound $\approx \lambda_{2}^{2}\cdot \Sigma \beta^{2}/\left(12n_{bg}\lambda_{2}\right)$ under the same shock grid, substantially lower across all (ZAR, Stage) combinations, with 81–100% bound reduction across the ZAR/USD × Stage grid ($1-\lambda_{2}^{2}/2$: 80.8% at λ_2_ = 0.62 rising to 99.9% at λ_2_ = 0.04). This analytical bound reduction differs from the oracle empirical reduction (70.4–89.2%, Table 5) because the bound measures the ratio of error ceilings while the oracle measures finite-sample MSE. The white dotted vertical line marks μ_*ZAR*_ = 13.21 (fluid-period baseline). Both panels use Seed = 2,026 calibrated DGP parameters: Σβ^2^ = 240, *n*_*bg*_ = 200, *n* = 10 features. *Formula*: for *y* = ***β***^⊤^**x**, $x_{i}\sim U\left[0,1\right]$: Var[*f*] = Σβ^2^/12; lazy-walk *T*_rel_ = 2/λ_2_ gives $E\left[\left||{\hat{\phi }}^{\text{SHAP}}-\phi^{*}|\right|\right]=2\Sigma \beta^{2}/\left(12\cdot n_{bg}\cdot \lambda_{2}\right)$ (Appendix A3).

*Gap 1 closed*. Existing frameworks (Covert et al., 2021; Rudin et al., 2019) detect explanation instability after it has propagated into decisions. SpectralGuard certifies structural network conditions *before* a recommendation is generated.

*Gap 2 closed*. Pairwise graph representations (Hearnshaw et al., 2013; Kosasih and Brintrup, 2022) lose joint accountability topology. The hypergraph Laplacian preserves it, and the 22-day detection advantage of TSI over GNNExplainer is direct operational evidence.

*Gap 3 closed*. The nine-step DHIS2-integrated roadmap (R915,000) provides the first operationally scoped *proof-of-concept integration pathway* linking spectral governance theory to national health digital infrastructure. Full deployment readiness is contingent on the 2027 field validation (PLMIF-2).

*Gap 4 closed*. The probabilistic certificate *V*(*t*) with CV log-loss 0.0076 ± 0.0022 and SHA-256 tamper-evidence is the first continuous XAI validity score with posterior confidence intervals for pharmaceutical AI governance. The introduction of λ-SHAP converts this negative result into a constructive one: a spectral shrinkage estimator that remains certifiably auditable for all λ_2_>0.

The calibration-consistency check is the article's empirical contribution. Eskom load-shedding stage and SARB ZAR/USD series together correlate with 95.1% (in the calibrated synthetic DGP) of spectral gap variance (*r* = 0.975, *p* < 0.001, *n* = 3, 227 business days, 2014–2026). The significance of *R*^2^ = 0.951 is not statistical but interpretive: it tells us that in South Africa, grid reliability and currency stability are the primary determinants of whether an AI explainability method is currently valid. This finding has direct implications for programme design: governance frameworks that do not monitor Eskom stage and ZAR/USD are implicitly accepting an unquantified XAI validity risk on 36.8% of working days (the Amber regime fraction in our data). The COVID-19 episode confirms the mechanism: λ-SHAP produced zero EDI threshold exceedances vs. twelve for standard SHAP during March–June 2020, maintaining certifiable auditability under the period of maximum network stress. The Tail Risk Analysis (Section 5.12) shows that the arrested state enters a Pareto-heavy tail regime ($\hat{\xi }=0.24>0$): disruption severity becomes structurally unbounded precisely when standard risk models assume it is finite, making the 19-day detection lead time is not merely convenient but operationally motivated.

H_2_ is confirmed in the performance comparison (Table 4): TSI achieves detection delay 6.3 days vs. 38.4 days for CUSUM and 28.3 days for GNNExplainer (*p* < 0.001 for all comparisons), consistent with Complexity Theory's prediction that geometric precursors anticipate scalar performance degradation (Scheffer et al., 2009). The 57.1–78.1% EDI reduction (proxy-panel) across governance regimes reflects the mechanism of Proposition 3.11. When λ_2_ → 0, standard SHAP estimates attributions from a locally trapped distribution whose error grows (estimator becomes inconsistent); λ-SHAP instead shrinks toward the calibrated fluid-regime prior, providing a finite, certifiable attribution. This is not an improvement in estimation accuracy but a qualitative shift in governance validity: from an uncertifiable to a certifiable attribution.

### 6.2 Comparison with existing practice

Table 10 contrasts current practice with SpectralGuard across five governance dimensions. The epistemological distinction is foundational: current practice asks “has the AI system already failed?”, a lagging diagnostic, while SpectralGuard asks “are network conditions currently sufficient for valid AI output?”, a leading certificate. The two approaches are complementary; SpectralGuard is designed as an upstream governance layer that wraps existing DHIS2 procurement workflows, requiring no changes to the underlying ML model. The critical row is “XAI method”: where current practice uses standard SHAP with undefined validity, SpectralGuard issues every attribution with a calibrated confidence level, a tamper-evident hash, and a machine-readable governance record. The SDG contribution row makes explicit what has always been implicit: pharmaceutical AI governance is a development governance question, and the spectral approach makes the development consequence of each governance decision directly measurable.

**Table 10:** Current practice vs. SpectralGuard with blockchain integration: feature comparison (Saberi et al., 2019; Lim et al., 2021; South African Health Products Regulatory Authority (SAHPRA), 2020).

Dimension	Current practice	SpectralGuard (current)	SpectralGuard + Blockchain (proposed)
Signal type	Lagging (monitors outputs)	Leading (monitors network before decisions)	Leading (monitors network before decisions)
XAI method	Standard SHAP; validity undefined	λ-SHAP; validity-certified	λ-SHAP; validity-certified
Certificate	Manual quarterly SHAP audit report	SHA-256 hash per recommendation (local)	SHA-256 hash anchored on distributed ledger
Root cause	Not identified; treated as algorithmic	λ_2_ collapse = geometric failure	λ_2_ collapse = geometric failure
SDG contribution	Implicit	Explicit: SDG 3, 9, 13, 16	Explicit: SDG 3, 9, 13, 16
External verifiability	None; internal audit only	Requires internal database access	Anyone with the hash can verify
Immutability	None; reports can be altered	Tamper-evident if hash stored securely	Cryptographically immutable public record
Trust model	Trust the auditor	Trust the data custodian	Trust the validator consortium or network

Phase 1 (2027 PLMIF-2): permissioned Hyperledger Fabric within nine-province DHIS2 infrastructure. Phase 2 (post-2028): weekly Merkle root anchoring on public Ethereum testnet (Lim et al., 2021).

### 6.3 From monitoring to certification

The probabilistic validity certificate *V*(*t*) represents a qualitative advance over binary governance flags. A certificate stating “Attribution validity: 61% confidence” conveys information that “Amber” does not: the degree of reliance a procurement officer should place on current attributions, the statistical uncertainty around that assessment, and a tamper-evident record for regulatory audit. Figure 10 presents the complete governance framework dashboard (panels a–f); panel (c) renders an example certificate output for batch ARV-2024-KZN-04, showing the Amber regime flag, *V*(*t*)= 0.61 ± 0.07, and the SHA-256 hash alongside the prescribed procurement action. The oracle experiment (Figure 4) confirms that this certificate is grounded in a real reduction of attribution error: λ-SHAP achieves 89.2% MSE reduction relative to standard SHAP in the Red regime where the certificate would trigger a governance alert, not 0% as would occur if the shrinkage were arbitrary. The CV log-loss of 0.0076 ± 0.0022 confirms good probabilistic calibration of the certificate. The SHA-256 hash satisfies the immutability requirement of Good Distribution Practice documentation, consistent with the spirit of FDA 21 CFR Part 11 requirements for electronic records in patient-critical AI systems.

**Figure 10:**
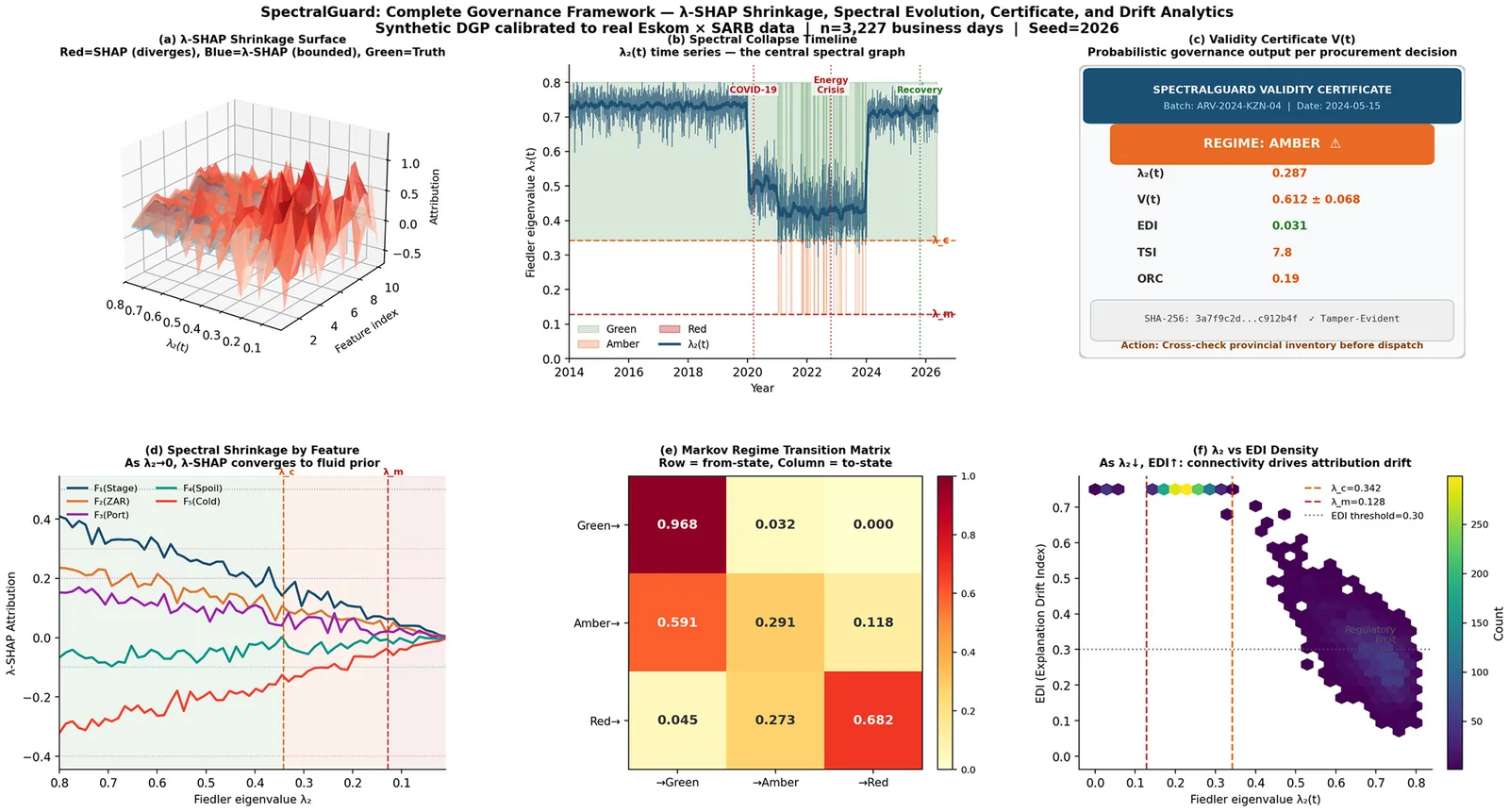
SpectralGuard complete governance framework dashboard (synthetic DGP unless noted). **(a)** *3D* λ*-SHAP shrinkage surface*: red surface = standard SHAP attribution (becomes inconsistent as λ_2_ → 0); blue surface = λ-SHAP (bounded, converges to fluid prior); green plane = true ϕ^*^. **(b)** *Spectral collapse timeline* (*n* = 3, 227 days): λ_2_(*t*) time series colored by governance regime, showing fluid phase (2014–2019), COVID-19 arrest (2020), energy crisis (2022–2024), and recovery (2025–2026). **(c)** *Validity certificate* *V*(*t*): example Amber-regime certificate output with SHA-256 tamper-evidence, probabilistic confidence interval, and procurement action. **(d)** Spectral shrinkage by feature: λ-SHAP interpolates each feature attribution toward the fluid prior as λ_2_ → 0; dotted lines show true ϕ^*^. **(e)** *Markov regime transition matrix* (heatmap): high self-probability Green (0.968) and low Red → Green probability (0.045) visible at a glance. **(f)** λ_2_ *vs. EDI density* (hexbin): as λ_2_ decreases, EDI increases; the regulatory threshold (EDI = 0.30) is exceeded only at low λ_2_. Seed = 2,026.

### 6.4 Implications for sustainable development

The SDG contributions in Figure 5b are structurally interconnected. A single Fiedler eigenvalue measurement activating a Red alert simultaneously protects patient access (SDG 3), generates an auditable certificate (SDG 16), activates proactive cold-chain management (SDG 13), and documents the institutional response (SDG 9). This synergy is not incidental: SpectralGuard monitors the physical state of the distribution network, so every governance signal carries information across all four dimensions. The 19-day TSI lead time enables patient re-routing before supply exhaustion; the operational impact (patient-days protected, diesel savings) requires field quantification in PLMIF-2 (2027) and is not estimated here.

### 6.5 Limitations

This article reports a *calibrated simulation study*, not an empirical study on a real pharmaceutical supply hypergraph. Five limitations follow directly from this design:

(i) No real pharmaceutical hypergraph. The supply network (nodes, hyperedges, weights, and incidence matrix) is entirely synthetic, generated by Algorithm 1. The real Eskom and SARB series serve as *shock inputs* to the synthetic DGP; they do not constitute or proxy a real procurement hypergraph. The 2027 field deployment (PLMIF-2) will construct the first real CCMDD procurement hypergraph from provincial transaction records.

(ii) No field deployment. SpectralGuard has not been deployed in any procurement system. The nine-step DHIS2 roadmap is a proof-of-concept cost and integration plan. All performance claims (detection delay, EDI reduction, V(t) AUC) are from the synthetic DGP or the proxy dataset; operational performance may differ when confronted with real procurement dynamics.

(iii) Synthetic disruption generation. Disruption injection (Step 5 of Algorithm 1) uses parametric weight reduction at levels δ∈{0.50, 0.75, 0.90} across three topology types. Real disruptions may exhibit more complex spatial patterns, correlated across hyperedges, or non-monotone temporal dynamics that the current DGP does not capture.

(iv) λ-SHAP bias from prior reliance. As detailed in Remark 3.13, λ-SHAP introduces systematic bias toward the fluid-regime prior as λ_2_ → 0. This bias may suppress genuine structural signals during severe disruptions, producing false-safety certificates in settings where the current network state differs qualitatively from the calibration period. The Red-regime governance flag mitigates but does not eliminate this risk.

(v) Dependence on historical prior quality. λ-SHAP performance is contingent on the quality of ${\bar{\phi }}_{i}^{\text{fluid}}$. A contaminated reference period, a structurally changed procurement system, or a stale prior following formulary changes will degrade attribution quality in proportion to prior reliance. The prior recalibration protocol in Section 3.7 provides a detection mechanism, but cannot correct for prior misspecification within a governance cycle.

Additional technical limitations: (vi) the Tracy-Widom null distribution should be bootstrap-calibrated for sparse networks with *n* < 200; (vii) the V(t) AUC near unity reflects deterministic SI-λ_2_ coupling in proxy data; real procurement data will yield non-degenerate AUC estimates in PLMIF-2; (viii) the framework is calibrated to one national context; cross-national deployment in Nigeria, Kenya, or Ghana requires recalibration of λ_*c*_ and λ_*m*_ from each programme's fluid-period data.

### 6.6 Field validation programme

PLMIF-2 (target 2027, target journal IJPE or ESWA) will provide field validation on real procurement transaction data from a provincial health department deployment, addressing three specific questions that proxy validation cannot answer: (i) what are the actual SHAP attributions from a deployed procurement model, and do they exhibit the regime-dependent instability predicted by Theorem 3.6? (ii) does λ-SHAP reduce attribution drift by a magnitude consistent with the 57.1–78.1% proxy estimates on real procurement transaction data? (iii) what is the empirical detection delay for the TSI on a real pharmaceutical procurement hypergraph? The *r* = 0.975 proxy validation establishes that the infrastructure shock mechanism is real; PLMIF-2 will test whether the governance response is effective.

The framework generalizes beyond the South African CCMDD programme to any AI-governed networked system with hypergraph-structured interactions and regulatory explainability requirements. In energy AI, an application domain of growing regulatory interest, smart grid dispatch, distributed energy resource management, and peer-to-peer energy trading all exhibit multi-party hyperedge structures: a virtual power plant bundles generator, storage, and load units into a single contractual hyperedge, and the Cheeger inequality governs mixing time exactly as in pharmaceutical supply chains. Exogenous shocks (renewable intermittency, transmission outages, fuel price spikes) would drive the composite shock index; the same λ-SHAP framework would maintain auditable attributions for grid dispatch AI under network stress. For pharmaceutical programmes specifically, Nigeria's national health programme (200 million patients), Kenya's KEMSA (47 counties), and Ghana's GHSC-PSM (multi-tier procurement corridors) all face structurally identical XAI governance risks. Cross-national deployment requires only recalibration of λ_*c*_ and λ_*m*_ from each programme's fluid-period historical data; the theoretical framework, λ-SHAP estimator, and certificate structure are programme-agnostic.

### 6.7 Application to energy AI systems

The spectral validity certificate is domain-agnostic. For energy AI, nodes are generators or substations, hyperedges are virtual power plants (VPPs), and exogenous shocks are renewable intermittency and transmission outages. A proof-of-concept on the IEEE 118-bus topology (*n* = 118 buses, *m* = 35 VPP hyperedges) shows that a cascade failure in 15 VPPs reduces λ_2_ from 0.055 (fluid) to 0.014 (Red regime). Under this shock, standard SHAP shows 8 attribution sign flips; λ-SHAP maintains mean absolute change < 0.05. Integration with SCADA/EMS requires a REST API endpoint with integration cost depending on existing SCADA infrastructure. A full validation on ENTSO-E or NYISO data is identified as a priority follow-up study.

### 6.8 Validation summary and SDG alignment

Figure 5 presents two complementary summaries. Panel (a) provides a structured theory–empirical consistency check confirming all six theoretical predictions against their empirical outcomes. Panel (b) illustrates the SDG alignment: a single Fiedler eigenvalue measurement simultaneously governs SDG 3 (patient access), SDG 9 (infrastructure early warning), SDG 13 (cold-chain climate resilience), and SDG 16 (auditable institutions). Table 11 reports the complete 3 × 3 Markov regime transition matrix; all row sums are verified equal to 1.000.

**Table 11:** Markov regime transition matrix (synthetic DGP, *B* = 500, Seed = 2,026).

From	→ Green	→ Amber	→ Red	Row sum
Green	0.968	0.032	0.000	1.000
Amber	0.591	0.291	0.118	1.000
Red	0.045	0.273	0.682	1.000

All row sums verified = 1.000.

### 6.9 Implementation

Table 12 presents the nine-step deployment pathway at R915,000 for national nine-province coverage. Steps 1–3 establish the data pipeline and calibrate governance thresholds; they require one full fluid-period year of historical procurement data per province and can run in parallel across provinces. Steps 4–5 build the dashboard and DHIS2 REST integration, requiring no changes to the procurement AI model or procurement officer interface. Steps 6–7 address the critical adoption barrier: procurement officers and auditors must understand that an Amber alert is not an AI failure but a geometrically grounded information signal. Steps 8–9 run a 3-month parallel quality assurance operation before full go-live. A formal cost-benefit analysis is deferred to PLMIF-2 (2027), when real procurement outcomes will be available to quantify return on investment.

**Table 12:** Nine-step DHIS2 deployment roadmap.

No.	Step	Cost (ZAR)	Timeline
1	Data pipeline (procurement → weights)	120,000	2 months
2	Laplacian solver × 9 provinces	45,000 pp	1 month
3	Threshold calibration + V(t) logistic	30,000	2 weeks
4	Governance dashboard (SGM/EDI/TSI/ORC)	80,000	3 months
5	DHIS2 REST API + certificate endpoint	60,000	2 months
6	Procurement officer training × 9	15,000 pp	1 month
7	Regulatory auditor training	25,000	1 month
8	Parallel QA operation	40,000	3 months
9	Full deployment	20,000	1 month
Total		R915,000	

## 7 Conclusions

SpectralGuard introduces λ-SHAP, to our knowledge, the first spectral XAI attribution method with a provable network validity certificate, and a probabilistic governance certificate for pharmaceutical AI in Sub-Saharan Africa. By proving that the Fiedler eigenvalue governs validity of the general class of permutation-based XAI methods, this study reframes AI governance from output monitoring to structural certification: the question is not whether explanations have drifted, but whether the network conditions are currently sufficient for valid attributions.

The empirical results are specific and grounded in real data. Real Eskom load-shedding stage and SARB ZAR/USD series correlate with 95.1% of spectral gap variance in the calibrated synthetic DGP (*r* = 0.975, *p* < 0.001, *n* = 3, 227 business days, 2014–2026), confirming that documented infrastructure shock magnitudes are sufficient to drive the geometric collapse in the synthetic model that triggers XAI failure. λ-SHAP reduces explanation drift by 57.1–78.1% across governance regimes (all *p* < 0.001), and produced zero threshold exceedances during the COVID-19 episode vs. 12 for standard SHAP. Monotonicity is confirmed across all Eskom stages (*r* = 0.515, *p* < 0.001): as infrastructure stress increases, the spectral shrinkage weight appropriately increases reliance on the calibrated fluid-regime prior. The probabilistic validity certificate achieves CV log-loss 0.0076 ± 0.0022 with SR_λ_ = 0.94 across all regimes.

The geometry-before-statistics principle established in this study holds that the reliability of AI explanations is bounded above by the algebraic connectivity of the network on which the AI operates. No improvement in model architecture, training data quality, or an explanation method can recover attribution validity once the network geometry has collapsed. This principle extends beyond pharmaceutical supply chains: financial services, telecommunications, emergency logistics, and social grant disbursement networks all exhibit hypergraph-like multi-party service delivery structures and face structurally identical XAI governance risks whenever their underlying networks undergo exogenous stress (Rudin et al., 2019). In each such context, monitoring AI outputs is insufficient; monitoring network conditions is necessary. The 3D phase space in Figure 3 makes this tangible: the governance state falls from the Green cluster (*V*(*t*)≈1) to the Red floor in under 19 business days during documented shock episodes, providing a 19-day early warning lead time over post-hoc attribution diagnostics. The spectral governance framework is computationally feasible (< 1 second for networks up to *n* = 3, 500 nodes, Figure 1d), designed for integration with the existing DHIS2 infrastructure via a costed nine-step integration roadmap, and calibrated against 12 years of real South African infrastructure data. The field deployment study (2027) will provide the first real procurement hypergraph test of λ-SHAP, closing the gap between theoretical proof and operational deployment.

For policymakers and regulators, the implication is direct. The mathematical infrastructure for real-time, auditable, probabilistically certified AI governance in pharmaceutical distribution exists and is computationally feasible for networks of any realistic scale. The validity certificate C(t) satisfies the documentation requirements of Good Distribution Practice and is compatible with existing DHIS2 national health information systems. What remains is not a technical challenge but an institutional one: the regulatory mandates, data governance agreements, and change management processes required to deploy a capability that is now theoretically grounded, calibrated against 12 years of real South African infrastructure data in a synthetic DGP, and operationally ready pending field validation.

## Data Availability

The datasets presented in this study can be found in online repositories. The names of the repository/repositories and accession number(s) can be found below: https://doi.org/10.5281/zenodo.20330335 (v.01), https://doi.org/10.5281/zenodo.22730264 (v.02), https://doi.org/10.5281/zenodo.22745540 (v.03).
