# Supplementary material for: SpectralGuard: a Spectrally grounded λ-SHAP framework for XAI validity certification in pharmaceutical supply chain networks
Source: Front Artif Intell. 2026 Oct 23;9:1884880. doi: 10.3389/frai.2026.1884880 (PMC13739688; doi:10.3389/frai.2026.1884880)
Supplement: Supplementary file 1 [file Supplementary_file_1.pdf]

Supplementary Material

SpectralGuard: A Spectrally Grounded λ-SHAP Framework for XAI Validity Certification in Pharmaceutical Supply Chain Networks

Frontiers in Artificial Intelligence — Manuscript ID: 1884880 — doi: 10.3389/frai.2026.1884880

Moroke ND and Lebele MM (2026)

This Supplementary file contains the complete mathematical appendices referenced in the main manuscript. All equation, lemma, and theorem numbers below correspond to the Appendix numbering in the main text (Appendix 1 = Section A1–A4; Appendix 2 = Section A5 GPD derivation; Appendix 3 = Sections A5–A7 null distributions; Appendix 4 = spectral graph interpretation; Appendix 5 = reproducibility code and data availability).

A Complete Proof of Theorem 3.6 (Spectral XAI Validity)

The main text contains the four-step proof. This appendix provides the full mathematical expansions with all constants, edge cases, and auxiliary lemmas required for a self-contained verification.

A.1 Auxiliary Lemma: Spectral Gap and Escape Rate

Lemma A.1 (Conductance Escape Bound). *Let $S \subset \mathcal{V}$ be the connected component containing the query node and $\Phi(\mathcal{H})$ the hypergraph conductance. For any $t > 0$:*

$$\Pr\big(X_t \notin S \mid X_0 \in S\big) \;\le\; t \cdot \Phi(\mathcal{H}) \;\le\; t\sqrt{2\lambda_2}. \tag{A1}$$

Proof. Escape probability within t steps is bounded by $t \cdot \Phi(S)$ (union bound). The hypergraph Cheeger inequality gives $\Phi(\mathcal{H}) \le \sqrt{2\lambda_2}$ [?, ?]. Hence $\Pr(X_t \notin S) \le t\sqrt{2\lambda_2} \to 0$ as $\lambda_2 \to 0$ for fixed t: the walk is trapped. □

A.2 Full Derivation: Total Variation Divergence

By Lemma Lemma A.1, after T_{dec} steps:

$$\Pr\big(X_{T_{\text{dec}}} \in S \mid X_0 \in S\big) \ge e^{-\lambda_2 T_{\text{dec}}}. \tag{A2}$$

The probability mass remaining in S is at least $e^{-\lambda_2 T_{\text{dec}}}$, so the walk has not mixed to stationarity over $\mathcal{V}$. The total variation distance between the empirical distribution $\hat{P}_S$ and the global distribution $P_{\mathcal{V}}$ satisfies:

$$\begin{aligned} d_{\text{TV}}(\hat{P}_S, P_{\mathcal{V}}) &= \tfrac{1}{2}\sum_x |\hat{P}_S(x) - P_{\mathcal{V}}(x)| \\ &\ge \Pr(X_{T_{\text{dec}}} \in S) - \pi(S) \\ &\ge e^{-\lambda_2 T_{\text{dec}}} - \pi(S), \end{aligned} \tag{A3}$$

where $\pi(S) = \mathrm{vol}(S)/\mathrm{vol}(\mathcal{H})$. When $\lambda_2 \to 0$, the right-hand side approaches $1 - \pi(S)$. If S is a proper subset ($\pi(S) < 1$), this quantity is strictly positive and bounded away from zero, confirming $d_{\mathrm{TV}} \to 1 - \pi(S) > 0$ for proper S.

A.3 Full Derivation: Upper Bound M_Φ/λ_2

The estimator $\hat{\phi}_i$ of any $\Phi \in \mathcal{P}$ marginalises f over N permutation samples drawn from $\hat{P}_S$. Let $Z_j = f(\mathbf{x}^{(j)}) - \mathbb{E}_{P_\mathcal{V}}[f]$ be the j-th centred evaluation. Since $\hat{P}_S \neq P_\mathcal{V}$, the Z_j are correlated with decorrelation time $\tau_{\mathrm{rel}} = 2/\lambda_2$. By the mixing-time variance inflation theorem (Levin and Peres [?], Theorem 12.19):

$$\mathrm{Var}\left[\frac{1}{N}\sum_{j=1}^{N} Z_j\right] \leq \frac{\tau_{\mathrm{rel}}}{N} \cdot \sigma_f^2 = \frac{\sigma_f^2}{N\lambda_2}, \tag{A4}$$

where $\sigma_f^2 = \mathrm{Var}_{P_\mathcal{V}}[f] \leq \|f\|_{\mathrm{Lip}}^2$ is bounded for Lipschitz f. The bias term from topology trapping is:

$$\mathrm{Bias}(\hat{\phi}_i) = \mathbb{E}_{\hat{P}_S}[f] - \mathbb{E}_{P_\mathcal{V}}[f] \leq \|f\|_{\mathrm{Lip}} \cdot d_{\mathrm{TV}}(\hat{P}_S, P_\mathcal{V}). \tag{A5}$$

Combining bias and variance via the standard decomposition, and using $d_{\mathrm{TV}} \leq 1$:

$$\begin{aligned} \mathbb{E}\|\hat{\phi}_i - \phi_i^*\|_{L_1} &\leq \mathrm{Bias}(\hat{\phi}_i) + \sqrt{\mathrm{Var}(\hat{\phi}_i)} \\ &\leq \|f\|_{\mathrm{Lip}} + \frac{\|f\|_{\mathrm{Lip}}}{\sqrt{N\lambda_2}} \\ &\leq \frac{C_\Phi \|f\|_{\mathrm{Lip}}}{\lambda_2} =: \frac{M_\Phi}{\lambda_2}, \end{aligned} \tag{A6}$$

where C_Φ is a finite method-specific constant. Note that the factor $\sqrt{2/N} \to 0$ as $N \to \infty$, so $C_\Phi(N)$ is bounded uniformly over $N \geq 1$ (specifically, $\sup_{N\geq 1} C_\Phi(N) < \infty$ since $\sqrt{2/N} \leq \sqrt{2}$ for all $N \geq 1$). Proposition 3.12 therefore holds uniformly in N, and the bound improves as N increases. $M_\Phi = C_\Phi\|f\|_{\mathrm{Lip}}$ is finite for any $\lambda_2 > 0$. As $\lambda_2 \to 0$, $M_\Phi/\lambda_2 \to \infty$.

A.4 Edge Case: $\lambda_2 = 0$ Exactly

When $\lambda_2 = 0$ exactly, $\mathcal{H}$ is disconnected. The background distribution $\hat{P}_S$ is supported entirely on the connected component containing the query, so $d_{\mathrm{TV}} = 1 - \pi(S)$. If $\pi(S) < 1$ (proper component), the attribution error is bounded below by $(1 - \pi(S))\|f\|_{\mathrm{Lip}} > 0$, which is finite but nonzero. The upper bound M_Φ/λ_2 is undefined (infinite) at $\lambda_2 = 0$; this is consistent with the theorem's statement that the upper bound grows without bound as $\lambda_2 \to 0$ (while the lower bound converges to a finite positive constant). In practice, the DGP clips $\lambda_2 \geq 0.01$ (Table Table 2) to avoid degenerate disconnected realisations.

B GPD-Spectral Tail Coupling Theorem and Proof

Theorem B.1 (GPD-Spectral Tail Coupling)**.** *Let $Z_t = -\log \lambda_2^{(t)}$ be the log-transformed spectral risk index and $u_0 = -\log \lambda_m$ the governance arrest threshold. For exceedances above u_0, the conditional excess distribution converges to a Generalised Pareto distribution (GPD):*

$$\Pr\big(Z_t - u_0 > x \mid Z_t > u_0\big) \to 1 - \left(1 + \frac{\xi x}{\sigma}\right)^{-1/\xi}, \quad x \geq 0, \tag{A7}$$

with shape $\hat{\xi} = 0.24 > 0$ (arrested state) versus $\hat{\xi}_{\text{fluid}} = -0.058 < 0$ (fluid state). The null hypothesis $H_0 : \xi_{\text{fluid}} = \xi_{\text{arrested}}$ is rejected by a likelihood ratio test ($\Lambda = 427.3$, $p < 0.001$, $df = 2$).

Proof. The Pickands-Balkema-de Haan theorem [?, ?] guarantees that for any distribution F in the maximum domain of attraction, there exist normalising functions $\sigma(u)$ and ξ such that the conditional excess distribution converges to the GPD as the threshold $u \to \infty$. We verify two conditions for the arrested state: (i) Z_t is regularly varying with index $\alpha = 1/\xi$ when $\xi > 0$, which follows from the Pareto tail of the mixing-time distribution as $\lambda_2 \to 0$; (ii) the threshold $u_0 = -\log \lambda_m \approx 2.06$ is in the upper tail of the arrested-state distribution, confirmed by the mean excess plot. The likelihood ratio statistic is:

$$\Lambda = 2\big[\ell(\hat{\xi}_A, \hat{\sigma}_A) + \ell(\hat{\xi}_F, \hat{\sigma}_F) - \ell(\hat{\xi}_0, \hat{\sigma}_0)\big] = 427.3, \tag{A8}$$

where $\ell(\cdot)$ denotes the GPD log-likelihood, subscripts A, F denote arrested and fluid MLE estimates, and subscript 0 denotes the constrained equal-ξ estimate. Under H_0, $\Lambda \sim \chi^2_2$; the critical value at $\alpha = 0.001$ is 13.8, so $\Lambda = 427.3$ rejects H_0 with overwhelming evidence. The governance implication is that $\xi > 0$ in the arrested state places disruption severity in the domain of Pareto-heavy tails, where finite safety-stock calculations based on bounded-tail assumptions are invalid. □

C Governance Metric Null Distributions

A.5 SGM: Tracy-Widom Calibration

The Spectral Governance Metric $T_{\text{SGM}} = (\hat{\lambda}_2 - \mu_{TW})/\sigma_{TW}$ follows the Tracy-Widom distribution TW_1 under the null of a Wigner random matrix [?]. The parameters μ_{TW} and σ_{TW} are computed from the fluid-period Fiedler eigenvalue series: $\mu_{TW} = \hat{\mu}_{\text{fluid}}$, $\sigma_{TW} = \hat{\sigma}_{\text{fluid}}$. For sparse hypergraphs with $n < 200$, the Tracy-Widom approximation is imprecise; in such cases we recommend bootstrap calibration: draw $B = 2{,}000$ fluid-period subsamples of size n, compute $\hat{\lambda}_2$ for each, and use the 5th and 95th percentiles as the alert thresholds.

A.6 EDI: Bootstrap Threshold Calibration

The Explanation Drift Index threshold $\tau_{\text{EDI}} = 0.30$ is derived from the fluid-period distribution of $\text{EDI}_f(t)$ values. The bootstrap procedure: (i) draw $B = 2{,}000$ samples of size n_{fluid} from the fluid-period EDI series; (ii) compute the 95th percentile of each sample; (iii) set τ_{EDI} at the mean of the bootstrap 95th percentile distribution. This ensures that the threshold corresponds to the level exceeded on 5% of days under normal programme operation.

A.7 TSI: Persistent Homology Computation

The Topological Stability Index integrates the first Betti number $\beta_1(\varepsilon, t)$ over a filtration range $[\varepsilon_{\min}, \varepsilon_{\max}]$:

$$\text{TSI}(t) = \int_{\varepsilon_{\min}}^{\varepsilon_{\max}} \beta_1(\varepsilon, t)\, d\varepsilon. \tag{A9}$$

The Rips complex is built on the node set $\mathcal{V}$ with pairwise distances $d(v, u) = 1 - w_{vu}/w_{\max}$ (normalised inverse weight). The filtration parameter ε ranges from 0 to 1 in $K = 100$ steps. Computationally, β_1 is computed by the GUDHI library [?] via the Vietoris-Rips persistent homology pipeline. The TSI null distribution uses a permutation test: $B = 500$ random weight permutations on the fluid-period hypergraph generate the null TSI distribution, with alert threshold at the 95th percentile.

D Spectral $\lambda_2(t)$ Time Series: The Spectral Graph

A direct answer to the question "what is spectral about this paper?" is provided by the $\lambda_2(t)$ series itself, which constitutes the paper's central spectral object. Figure Figure 2(b) in the main text shows this series. Algebraically, $\lambda_2(t)$ is the second smallest eigenvalue of the normalised hypergraph Laplacian $\mathbf{L}(t)$ (Definition Definition 3.2), computed at each t via the ARPACK sparse eigensolver applied to the $n \times n$ matrix $\mathbf{L}(t) = \mathbf{I} - \mathbf{D}_v^{-1/2}(t)\mathbf{H}(t)\mathbf{W}(t)\mathbf{D}_e^{-1}(t)\mathbf{H}^\top(t)\mathbf{D}_v^{-1/2}(t)$. The spectral graph in Figure 3(b) shows three structurally distinct phases: (i) the fluid regime (2014–2019), where $\lambda_2 \in [0.55, 0.80]$ and the network mixes globally within the daily decision horizon; (ii) the arrested state (COVID-19 2020, energy crisis 2022–2024), where λ_2 drops below $\lambda_c = 0.342$ into the Amber zone and below $\lambda_m = 0.128$ into the Red zone, and the information propagation network fractures into poorly connected components; and (iii) the recovery period (2025–2026), where Stage 0 load-shedding allows λ_2 to return toward the fluid baseline. The spectral collapse during the COVID-19 episode (March–June 2020) is the empirical anchor for the calibration: $\lambda_2(t)$ in the synthetic DGP enters the Red regime during the same shock window that real supply disruptions were documented. The causal link between spectral collapse and procurement failure is the hypothesis of PLMIF-2, not an established finding of this paper.

E DGP Reproducibility and Code

All results are reproducible with `Seed=2026`. The full DGP code, oracle experiment, and ablation study scripts are available at [repository withheld for blind review]. The repository contains: `spectralguard_dgp.py` (Algorithm Algorithm 1 implementation); `oracle_experiment.py` (Table Table 5 replication); [script names withheld for blind review] (Tables Table 1, Table 6, and Table 7 replication scripts available upon acceptance).

Key dependencies: Python 3.10, NumPy 1.24, SciPy 1.10 (ARPACK eigensolver), scikit-learn 1.2 (logistic regression for $V(t)$), GUDHI 3.7 (persistent homology for TSI). All dependencies are listed in `requirements.txt` in the repository.

[Revised: M13 — references corrected and expanded]
